# Double Thionation of 4‐Dimethylaminophthalimide Leads to the Development of a Highly Effective Photosensitizer

**DOI:** 10.1002/chem.71137

**Published:** 2026-05-18

**Authors:** Chris Acquah, Eric Lee, Sourav Kanti Seth, Reshma Mathew, Rubej R. Khan, Steffen Jockusch, Shudan Yang, Han Xiao, Carlos E. Crespo‐Hernández

**Affiliations:** ^1^ Department of Chemistry Case Western Reserve University Cleveland Ohio USA; ^2^ Center for Photochemical Sciences Bowling Green State University Bowling Green Ohio USA; ^3^ Department of Chemistry Rice University Houston Texas USA; ^4^ Department of Biosciences Rice University Houston Texas USA; ^5^ Department of Bioengineering Rice University Houston Texas USA

**Keywords:** intersystem crossing, photodynamic therapy, singlet oxygen generation, thionation

## Abstract

Transforming existing biocompatible fluorophores through thionation presents a compelling strategy to modulate their photodynamic properties while circumventing the limitations associated with heavy atoms, such as metals or halogens. This approach integrates sulfur atoms that enhance intersystem crossing (ISC) to the triplet state via spin‐orbit coupling, positioning these photosensitizers as suitable candidates for photodynamic therapy applications. In this study, we investigate the photophysical properties and excited‐state dynamics of 4‐dimethylaminophthalimide (DMAP) and its thionated derivative, thio‐4‐dimethylaminophthalimide (SDMAP). DMAP exhibits a fluorescence quantum yield of 17%, demonstrating significant fluorescence emissions. In contrast, SDMAP shows negligible fluorescence due to enhanced ISC within 1.0 ± 0.2 ps, which facilitates the rapid triplet state population, with a lifetime of 3.40 ± 0.05 µs under argon‐saturated conditions. Notably, the triplet state of SDMAP generates singlet oxygen with a quantum yield of 0.83 ± 0.05, highlighting its high potential for photodynamic therapy. In comparison, DMAP does not produce singlet oxygen and undergoes radiative and nonradiative decay to the ground state within 5 ± 1 ns. These findings underscore the profound impact of thionation, effectively converting DMAP from a relatively fluorescent molecule into a highly efficient photosensitizer for photocatalysis and deep‐tissue photodynamic therapy applications.

## Graphical abstract

We report on the photophysical properties and electronic relaxation mechanisms of 4‐dimethylaminophthalimide (DMAP) and thio‐4‐dimethylaminophthalimide (SDMAP). While DMAP is fluorescent, SDMAP shows negligible fluorescence due to ultrafast intersystem crossing to a long‐lived triplet state that decays in 3.4 ± 0.05 µs. SDMAP efficiently generates singlet oxygen with a high quantum yield of 0.83 ± 0.05. These findings underscore the profound impact of thionation on DMAP's photophysical properties, effectively converting it from a relatively fluorescent molecule into a highly efficient photosensitizer for photocatalysis and deep‐tissue photodynamic therapy applications.

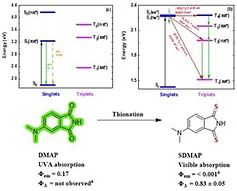

## 1 Introduction

Thionation of heavy‐atom‐free photosensitizers (HAF‐PSs) is an emerging strategy to enhance the photophysical properties of photosensitizing molecules without relying on heavy atoms like metals or halogens [1, 2, 3, 4, 5, 6, 7, 8, 9, 10, 11, 12, 13, 14, 15, 16, 17, 18, 19, 20, 21, 22, 23, 24, 25, 26, 27, 28]. Typically, heavy atoms are introduced into molecules to increase spin‐orbit coupling, a process that facilitates the transition from a singlet excited state to a triplet excited state [29, 30, 31, 32, 33]. However, heavy atoms often come with drawbacks such as high costs of synthesis, elevated dark cytotoxicity, limited biocompatibility, and short triplet state lifetimes [1, 4, 11, 34, 35, 36, 37, 38]. Thionation, which involves replacing oxygen atoms with sulfur in carbonyl groups, offers a way to enhance intersystem crossing (ISC) while maintaining the advantageous properties of HAF‐PSs, such as reduced dark cytotoxicity and high stability [1, 2, 3, 4, 5, 6, 7, 8, 9, 10, 11, 12, 13, 14, 15, 35, 39]. The significance of thionation lies in its ability to broaden the applicability of organic molecules in biomedical and energy‐related fields. HAF‐PSs modified via thionation exhibit improved triplet‐state yields because of an increase in ISC, which are essential for photocatalysis and for the effective generation of singlet oxygen that is crucial in PDT [1, 2, 3, 4, 5, 6, 7, 8, 9, 10, 11, 12, 13, 14, 15, 16, 17, 18, 19, 20, 21, 22, 23, 24, 25, 26, 27, 28].

Phthalimides, a class of π‐deficient heterocyclic compounds, have been explored for their photochemical properties and biological activities [40, 41]. Their derivatives show considerable potential as DNA‐targeting agents, anticancer, and anti‐inflammatory compounds. These compounds have also found use in various applications, including medicinal chemistry and pharmaceuticals, due to their impressive biological activities [40, 42]. Additionally, structural modifications to the imide moieties have produced derivatives with excellent chromophoric and fluorophoric properties, including high molar absorptivity, photostability, and low dark toxicity; qualities that align with the requirements for effective photosensitizers [40, 43, 44]. Specifically, thionation of phthalimides results in significant changes to their photophysical properties, notably enhancing triplet‐state population, improving ISC efficiency, and extending light absorption into longer wavelengths [11, 35, 40, 43, 45, 46].

**SCHEME 1:**
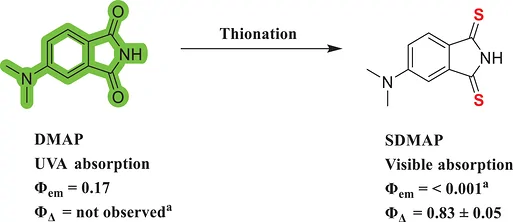
The structures of 4‐dimethylaminophthalimide (DMAP) and its thionated analog, thio‐4‐dimethylaminophthalimide (SDMAP) (see a: ref. [1]).

Scheme 1 presents two phthalimide‐based derivatives: 4‐dimethylaminophthalimide (DMAP) and its thionated analog, thio‐4‐dimethylaminophthalimide (SDMAP). DMAP has been reported by Vázquez and co‐workers as an environment‐sensitive fluorescent probe [47]. Additionally, SDMAP has demonstrated both biocompatibility and low dark cytotoxicity in cancer cells and 3D tumor spheroids [1]. However, a detailed understanding of their electronic relaxation mechanisms and photophysical properties is still lacking. In this contribution, steady‐state absorption and emission spectroscopy, femtosecond broadband transient absorption, nanosecond to microsecond transient absorption spectroscopy, and quantum chemical calculations are combined to investigate the excited‐state dynamics and electronic relaxation pathway of DMAP compared to its thionated analog, SDMAP, in acetonitrile (ACN).

## 2 Results and Discussion

### 2.1 Steady‐State Spectroscopy

**FIGURE 1:**
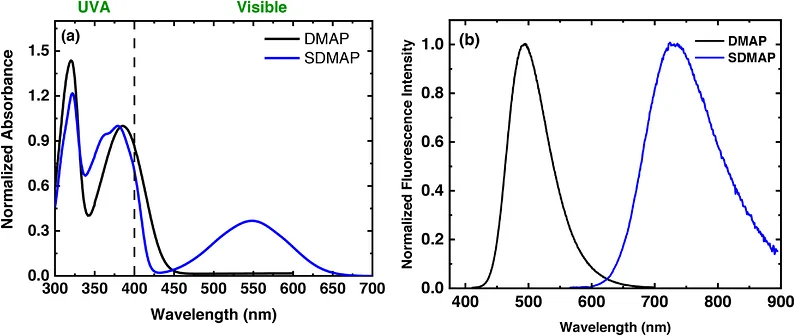
(a) Normalized absorption spectra of DMAP (black) and SDMAP (blue) in ACN. (b) Steady‐state fluorescence emission spectra of DMAP (black) and SDMAP (blue) following excitation at 400 and 550 nm for DMAP and SDMAP, respectively.

Figure 1 presents the normalized absorption and fluorescence emission spectra of DMAP and SDMAP in ACN. The absorption spectrum for DMAP shows two distinct maxima at approximately 320 and 385 nm, while SDMAP's spectrum exhibits maxima at 321, 378, and 548 nm. Upon double thionation of the succinimide ring in DMAP, a significant red shift of up to 160 nm is observed, extending absorption further into the visible region of the electromagnetic spectrum. This red shift aligns with prior steady‐state studies for other thionated HAF‐PSs [1, 2, 3, 4, 5, 6, 7, 8, 9, 10, 11, 12, 13, 14, 15, 48] and is further supported by quantum chemical calculations at the TD‐PBE0‐D3BJ/CPCM/def2‐TZVPD//B3LYP_G_‐D3BJ/CPCM/def2‐TZVPD level of theory in ACN (Figure S1a). Upon excitation at 400 nm, DMAP displays moderate emission centered around 494 nm in ACN. Under N_2_‐saturated conditions (Figure S2a), the emission intensity increases slightly (by 12% compared to ambient conditions), supporting the fluorescent nature of the emission. In contrast, under O_2_‐saturated conditions, the emission intensity decreases by 30%, consistent with the quenching effect of O_2_ on fluorescence. This behavior aligns with the relatively small Stokes shift (∼100 nm between the absorption and emission maxima), indicating minimal energy loss (Figure S2a). A quantum yield of 0.17 was obtained in ACN using Quinine Sulphate as a reference standard [49].

In contrast, SDMAP exhibited minimal emission at around 730 nm in ACN. The emission bands remained unchanged under both O_2_‐ and N_2_‐saturated conditions (Figure S2b). Notably, the excitation spectra recorded at the emission maxima for both DMAP and SDMAP closely align with the steady‐state absorption spectra, confirming that the emission is intrinsic to the compounds and not due to putative impurities (see Figure S2c,d). Additionally, the E_0,0_ energies of the excited singlet states of both molecules are estimated using the normalized excitation and fluorescence emission spectra (Figure S1b,c). The E_0,0_ energies of 2.80 and 1.92 eV are obtained for DMAP and SDMAP in ACN, respectively.

### 2.2 Quantum Chemical Calculations

All the calculations for DMAP and SDMAP were performed using ORCA 6.0.1 electronic structure software. The ground state optimizations for each molecule were conducted using density functional theory (DFT) [50] calculations at the B3LYP_G_‐D3BJ/CPCM/def2‐TZVPD level of theory. Subsequently, vertical excitation energies (VEE) and SOC were calculated at the TD‐PBE0‐D3BJ/CPCM/def2‐TZVPD level of theory in ACN. The PBE0 [51] functional was chosen for all time‐dependent calculations due to its superior accuracy in modeling the absorption of the molecules compared to other functionals tested, such as CAM‐B3LYP [52] and X3LYP [53] (Tables S1 and S2). Tables 1 and 2 present the energies and the oscillator strengths for the three lowest singlet and four lowest triplet states of DMAP and SDMAP at the FC region in ACN. Additionally, the percentage contributions of the single electron transitions and the Kohn‐Sham orbitals for both molecules are illustrated in Tables S3, S4, Figures S3, and S4, respectively.

**TABLE 1:** Vertical excitation energies (eV), oscillator strength, and energy gaps (eV) between the singlet and triplet excited states using the optimized FC geometry for DMAP and SDMAP computed at the TD‐PBE0‐D3BJ/CPCM/def2‐TZVPD//B3LYP_G_‐D3BJ/CPCM/def2‐TZVPD level of theory in ACN.

DMAP				SDMAP			
State	Electronic character	Energy (eV)	Oscillator strength	State	Electronic character	Energy (eV)	Oscillator strength
S_1_	ππ*	3.23	0.18	S_1_	nπ*	2.27	0.00
S_2_	nπ*	4.17	0.00	S_2_	ππ*	2.28	0.23
S_3_	ππ*	4.26	0.13	S_3_	nπ*	2.58	0.00
T_1_	ππ*	2.37		T_1_	ππ*	1.51	
T_2_	ππ*	3.35		T_2_	nπ*	1.98	
T_3_	nπ*	3.77		T_3_	ππ*	2.15	
T_4_	ππ*	3.87		T_4_	nπ*	2.29	
∆E(S_1_‐T_1_)		0.86		∆E(S_1_‐T_1_)		0.76	
∆E(S_1_‐T_2_)		−0.12		∆E(S_1_‐T_2_)		0.29	
∆E(S_1_‐T_3_)		−0.54		∆E(S_1_‐T_3_)		0.12	
∆E(S_2_‐T_1_)		1.8		∆E(S_2_‐T_1_)		0.77	
∆E(S_2_‐T_2_)		0.82		∆E(S_2_‐T_2_)		0.30	
∆E(S_2_‐T_4_)		0.30		∆E(S_2_‐T_4_)		−0.01	

**TABLE 2:** Calculated SOC constants for DMAP and SDMAP computed at the TD‐PBE0‐D3BJ/CPCM/def2‐TZVPD//B3LYP_G_‐D3BJ/CPCM/def2‐TZVPD level of theory using the FC geometries in ACN.

DMAP		SDMAP	
FC geometry	SOC (cm^−1^)	FC geometry	SOC (cm^−1^)
S_1_‐T_1_ (^1^ππ*‐ ^3^ππ*)	0.01	S_1_‐T_1_ (^1^nπ*‐ ^3^ππ*)	51
S_1_‐T_2_ (^1^ππ*‐ ^3^ππ*)	0.04	S_1_‐T_2_ (^1^nπ*‐ ^3^nπ*)	1.1
S_2_‐T_1_ (^1^nπ*‐ ^3^ππ*)	8.4	S_2_‐T_1_ (^1^ππ*‐ ^3^ππ*)	0.06
S_2_‐T_2_ (^1^nπ*‐ ^3^ππ*)	1.8	S_2_‐T_2_ (^1^ππ*‐ ^3^nπ*)	37
S_2_‐T_4_ (^1^nπ*‐ ^3^ππ*)	9.8	S_2_‐T_4_ (^1^ππ*‐ ^3^nπ*)	17
S_3_‐T_3_ (^1^ππ*‐ ^3^nπ*)	2.4	S_3_‐T_3_ (^1^nπ*‐ ^3^ππ*)	148

Based on the findings outlined in Table 1, it is proposed that DMAP predominantly populates its lowest energy singlet S_1_ (ππ*) state upon excitation at 400 nm (3.17 eV), supporting its fluorescent nature. This conclusion arises from the fact that the second‐lowest energy singlet state (S_2_) primarily exhibits an nπ* character with minimal oscillator strength. Furthermore, since the T_1_ state has a lower energy than S_1_, while the T_2_ state is nearly isoenergetic with the S_1_, ISC to the triplet manifold could effectively compete with radiative decay to the ground state. However, based on El Sayed's rules, ISC to the triplet manifold should not occur favorably between these states because the transition would involve states with the same type of orbitals. This is in good agreement with the calculated low SOC value of 0.01 and 0.04 cm^−1^ for S_1_‐T_1_ and S_1_‐T_2,_ respectively, reported in Table 2.

The lowest‐energy singlet excited state S_1_ of SDMAP has predominantly nπ* character and is isoenergetic with the S_2_ (ππ*) state. Given the characteristic ± 0.3 eV error associated with TD‐DFT calculations [13, 49, 54], both ^1^ππ* and ^1^nπ* states are predicted to be practically isoenergetic in the FC region of the PES. Considering the negligible oscillator strength usually associated with nπ* transitions, it is expected that most of the excited state population will initially reside in the ^1^ππ* state upon excitation at 602 nm (2.07 eV) in ACN. Furthermore, with four triplet states positioned lower than or equal in energy to the ^1^ππ* state, ISC to the triplet manifold is expected to be competitive.

**FIGURE 2:**
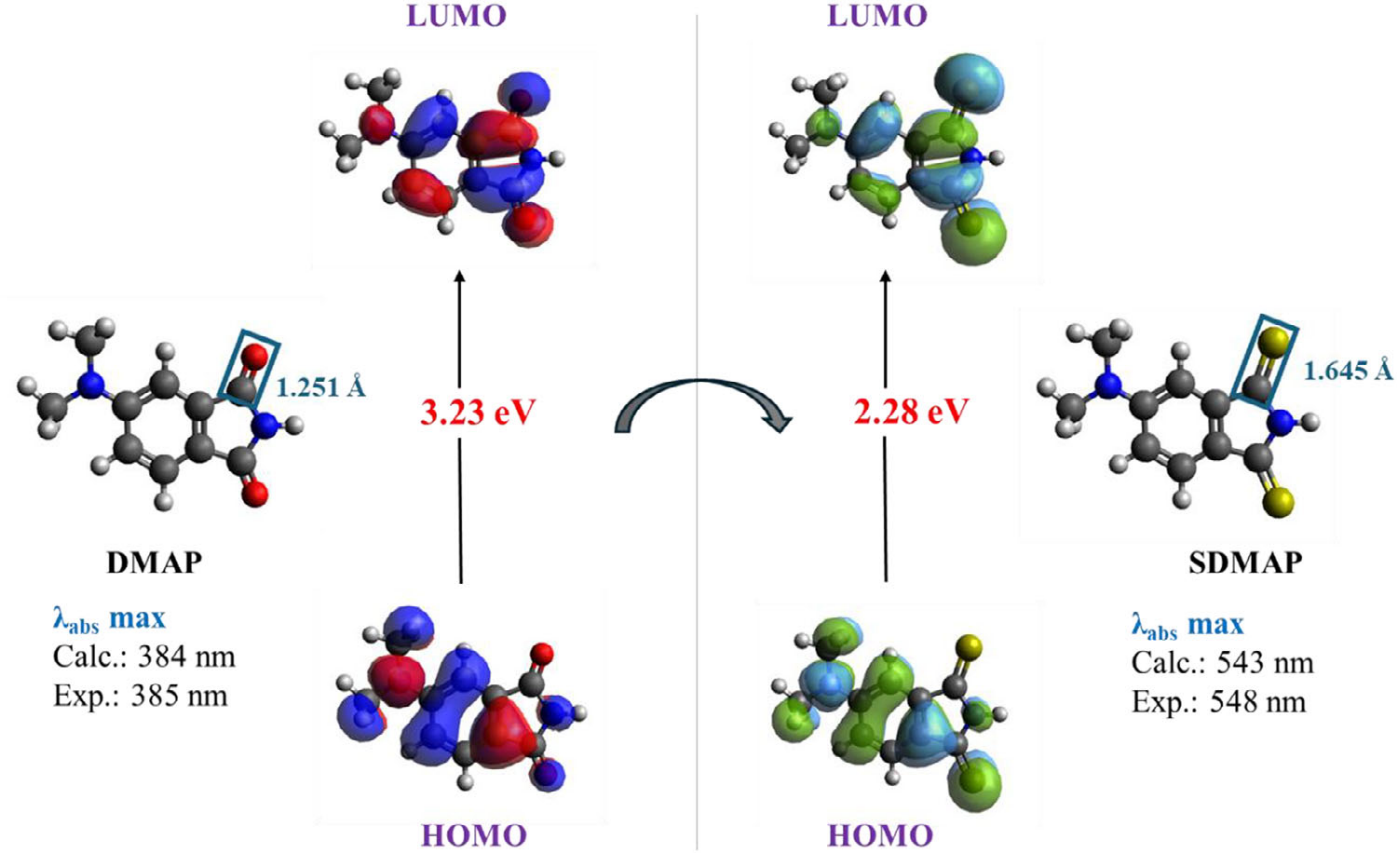
Frontier orbitals and energy gaps (eV) between DMAP and SDMAP computed at the TD‐PBE0‐D3BJ/CPCM/def2‐TZVPD//B3LYP_G_‐D3BJ/CPCM/def2‐TZVPD level of theory in ACN.

The small energy gaps (i.e., ∆E(S_1_‐T_1_) = 0.76, ∆E(S_1_‐T_2_) = 0.29, ∆E(S_2_‐T_2_) = 0.30, etc.) in Table 1 and large SOC results (i.e., S_1_‐T_1_ (^1^nπ*‐ ^3^ππ*) = 51 cm^−1^, S_2_‐T_2_ (^1^ππ*‐ ^3^nπ*) = 37 cm^−1^, etc.) presented in Table 2 suggest that multiple efficient ISC are likely in SDMAP. Particularly, ISC is predicted between the S_1_ state and the T_1_ and T_3_ states, between the S_2_ and S_3_ states with the T_2_ and T_3_ states, and between the near‐isoenergetic S_1_ and S_2_ states with the T_4_ (nπ*) state. This strong coupling between singlet and triplet states in the FC region is expected to enable efficient ISC to the triplet manifold through multiple pathways. Moreover, the weaker C═S bond compared to the C═O bond reduces the energy gap between the lowest unoccupied molecular orbital (LUMO) and the highest occupied molecular orbital (HOMO) (Figure 2). These adjustments to the frontier orbitals, combined with an increased density of states, make nonradiative pathways such as ISC more competitive than fluorescence emission [1, 3, 8, 35]. The enhanced ISC pathways predicted for SDMAP, relative to DMAP, highlight the critical role of thionation in facilitating ISC to the triplet manifold in these HAF‐PSs.

### 2.3 Electronic State Optimization

**FIGURE 3:**
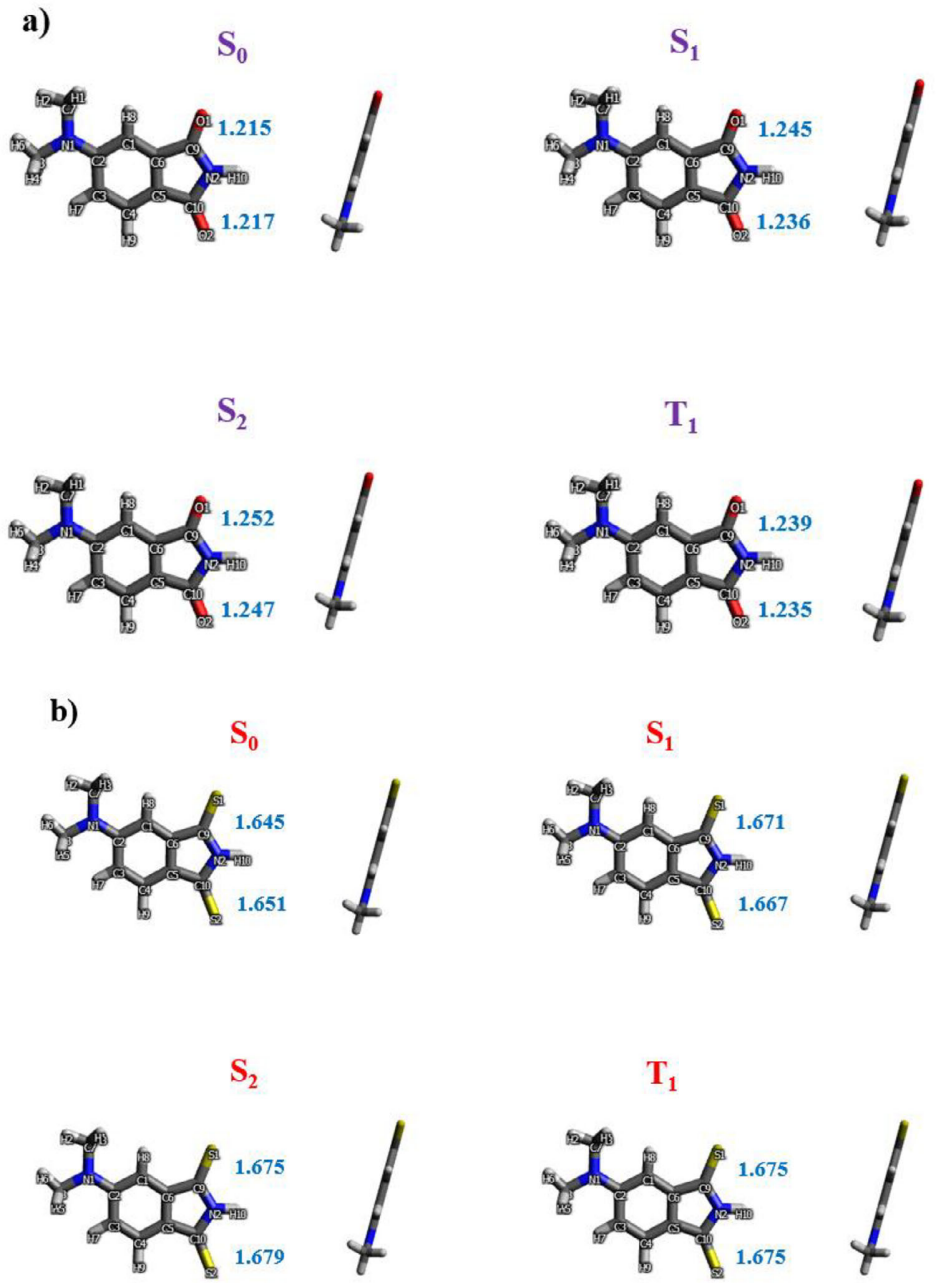
Optimized geometries of S_0_, S_1_, S_2_, and T_1_ excited states for (a) DMAP and (b) SDMAP at the TD‐PBE0/CPCM/def2‐TZVPD level of theory in ACN. The bond length values are in angstroms.

The electronic relaxation pathways of DMAP and SDMAP likely involve transitions through multiple excited electronic states. To investigate this in greater detail, the nuclear coordinates for the S_1_, S_2_, and T_1_ excited states of both compounds were optimized using the TD‐PBE0/CPCM/def2‐TZVPD level of theory in ACN. The VEE's and SOC's at the S_1_ and S_2_ minimum for both molecules are presented in the supplemental information (Tables S5–S8). Figure 3 illustrates the optimized geometries of DMAP and SDMAP, respectively. For both molecules, the lowest‐energy geometries of the S_1_, S_2_, and T_1_ states are predominantly planar in ACN, exhibiting minimal conformational deviations across the electronic states. A key structural distinction lies in the variation of the carbon‐oxygen and carbon‐sulfur bond lengths. For DMAP, the carbon‐oxygen (C9─O1) bond lengths are 1.215 (1.217), 1.245 (1.236), 1.252 (1.247), and 1.239 Å (1.235 Å) for the S_0_, S_1_, S_2_, and T_1_ states, respectively, with the values in parentheses corresponding to the C10─O2 bond length (Figure 3a).

In contrast, for SDMAP, the carbon‐sulfur (C9─S1) bond lengths are 1.645 (1.651), 1.671 (1.667), 1.675 (1.679), and 1.675 Å (1.675 Å) for the S_0_, S_1_, S_2_, and T_1_ states, respectively, with the values in parentheses indicating the corresponding bond lengths for C10─S2 (Figure 3b). These variations highlight subtle yet significant differences in the bonding characteristics of DMAP and SDMAP across their electronic states.

Additionally, the fluorescence energy was examined using the optimized S_1_ structures. For DMAP and SDMAP, the predicted vertical S_1_→S_0_ emission wavelengths are 460 and 688 nm, respectively. These values align well with the experimentally observed emissions of 494 nm for DMAP and 711 nm for SDMAP in ACN. Furthermore, the E_0,0_ values for DMAP and SDMAP in ACN are estimated to be 2.99 and 2.12 eV, respectively, which closely match the experimentally determined values of 2.80 eV for DMAP and 1.92 eV for SDMAP.

### 2.4 Femtosecond Broadband Transient Absorption Spectroscopy

**FIGURE 4:**
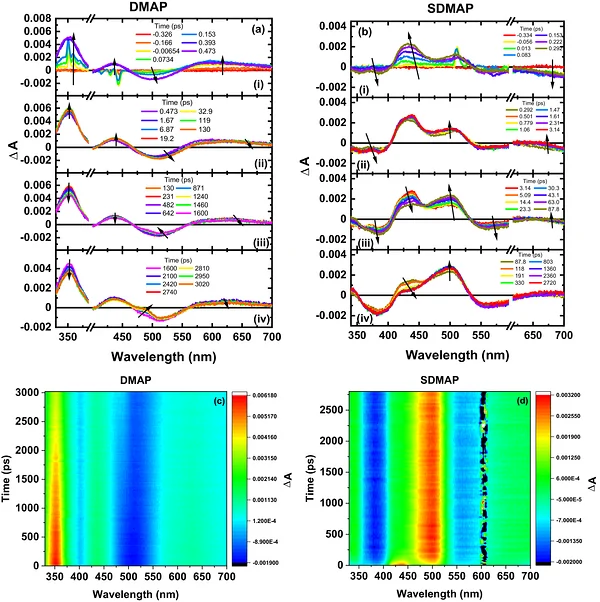
Spectral evolution of the transient absorption spectra of (a) DMAP and (b) SDMAP in ACN following excitation at 400 and 602 nm, respectively. The positive spike at ca. 513 nm in (b–i) corresponds to the stimulated Raman emission from the solvent. The breaks in panel (b) are covering the scattering of the pump reaching the detectors. (c–d) Representative contour plots of the TAS data for both compounds in ACN.

**FIGURE 5:**
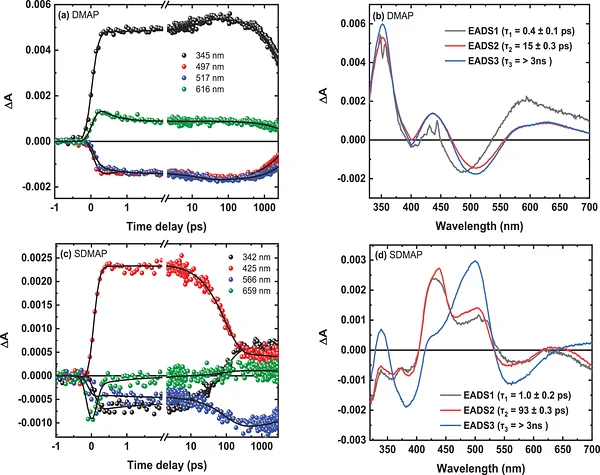
Representative kinetic traces and evolution‐associated difference spectra (EADS) of DMAP (a, b) and SDMAP (c, d) following excitation at 400 nm and 602 nm, respectively, in ACN, globally fit with a three‐component sequential model. The breaks on the x‐axis in panels (a) and (c) represent the change in scale from linear to logarithmic.

The excited‐state dynamics of DMAP and SDMAP were investigated through the analysis of time‐resolved absorption spectra. Figure 4 illustrates the transient absorption spectra of DMAP and SDMAP in ACN upon 400 and 602 nm excitation, respectively. Figure 5 presents representative decay traces with best‐fit curves and the evolution‐associated difference spectra for both compounds. Table 3 summarizes the lifetimes derived from a global fit analysis of the transient data for DMAP and SDMAP using a three‐component sequential kinetic model.

**TABLE 3:** Global lifetimes of DMAP and SDMAP following excitation at 400 and 602 nm, respectively, in ACN. All errors are reported as twice the standard deviation.

	DMAP	SDMAP
τ_1_	0.4 ± 0.1 ps	1.0 ± 0.2 ps
τ_2_	15.0 ± 0.3 ps	93.0 ± 0.3 ps
τ_3_	> 3 ns	> 3 ns

Upon excitation of DMAP at 400 nm, three distinct positive transient bands are observed within the cross‐correlation of the pump and probe beams, with maximum at 350, 435, and 605 nm. Additionally, a negative amplitude transient signal appears around 505 nm (Figure 4a–i), closely corresponding to the steady‐state fluorescence emission and, hence, attributed to stimulated emission. During the time delays from 0.47 to 130 ps, the transient species absorbing at 515 nm increases in intensity and experiences a slight red shift, while the positive band at 605 nm decreases in amplitude. Between approximately 130 ps and 1.6 ns, the positive bands at 350 and 435 nm begin to decay, alongside a reduction in the negative band at 505 nm. Notably, an isosbestic point is observed around 470 nm during this interval. Finally, from 1.6 ns to the end of the 3 ns probing regime (Figure 4a, iv), a new band emerges in the 475–503 nm range, resulting from the negative signal at 505 nm, coupled with a further decrease in the positive band at 605 nm.

Similarly, for SDMAP in ACN, following excitation at 602 nm, two positive ∆A bands are observed at 436 and 507 nm within the cross‐correlation of the pump and probe beams, along with a positive spike at approximately 510 nm, corresponding to stimulated Raman emission from the solvent. A negative ∆A band begins to grow between 350 and 420 nm, while a broad transient species emerges in the 600–700 nm range. Between approximately 3 and 87 ps, the initial positive band at 436 nm decays in amplitude, while the positive band at 505 nm slightly blue shifts and intensifies. The appearance of an isosbestic point around 457 nm suggests a state‐to‐state transition from the singlet to the triplet manifold through intersystem crossing. A detailed relaxation mechanism is mentioned below. The decrease in amplitude for the 436 nm band and a simultaneous rise in the intensity of the band ca. 505 nm are observed in the kinetic traces shown in Figure 5c. Simultaneously, the negative band between 350 and 400 nm slightly redshifts and increases in amplitude, while the broad transient species in the 600–700 nm region develops into a positive band with a maximum at 650 nm. Last, (Figure 4b, iv), after 87 ps and throughout the remainder of the 3 ns probing window, the positive band at 436 nm fully decays, while the positive band with a maximum at approximately 507 nm shows a slight increase in intensity. Additionally, the broad transient feature observed between 600 and 700 nm remains steady. The broadband transient absorption data for DMAP and SDMAP were well fitted using a three‐component sequential kinetic model. The global average lifetimes are provided in Table 3, and the evolution‐associated difference spectra (EADS) and representative kinetic traces from global and target analyses are displayed in Figure 5.

### 2.5 Nanosecond to Microsecond Transient and Singlet Oxygen Yield Experiments for DMAP and SDMAP

**FIGURE 6:**
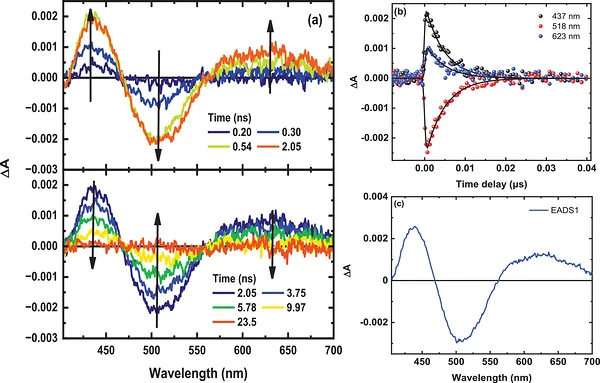
(a) Spectral evolution of the nanosecond transient absorption spectra of DMAP in ACN following excitation at 400 nm in ambient air conditions. (b) Representative kinetic decay traces and (c) evolution‐associated difference spectra obtained from global and target analyses of DMAP in ACN.

The transient absorption spectra discussed earlier were limited to a temporal window of 3 ns. To investigate the kinetics of DMAP and SDMAP with longer lifetimes, we employed nanosecond‐to‐microsecond transient absorption spectroscopy. As presented in Figure 6, following excitation of DMAP at 400 nm, the positive transient signals with maxima at 435 and 635 nm steadily decay to the baseline within approximately 15 ns, alongside the negative amplitude at 510 nm. This process provided a lifetime of 5 ± 1 ns under ambient air, and this lifetime remains the same for both O_2_ and N_2_‐saturated conditions (Figure S5, S6, and Table S9). This rapid decay indicates that the excited‐state dynamics of DMAP are dominated by radiative and/or nonradiative decay to the ground state rather than ISC or oxygen‐dependent processes. The unchanged lifetime across all conditions supports a negligible‐to‐no triplet state population, as triplet states are usually quenched by molecular oxygen. The absence of such effects confirms that DMAP undergoes purely singlet‐state decay.

**FIGURE 7:**
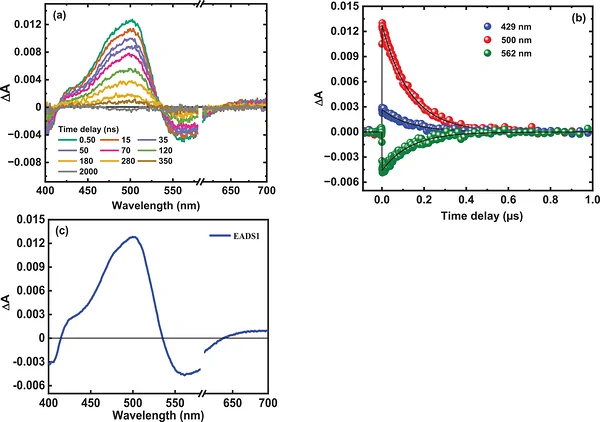
(a) Spectral evolution of the nanosecond transient absorption spectra of SDMAP in ACN following excitation at 600 nm in ambient air conditions. (b) Representative kinetic decay traces and (c) evolution‐associated difference spectra obtained from global and target analyses of SDMAP in ACN.

**FIGURE 8:**
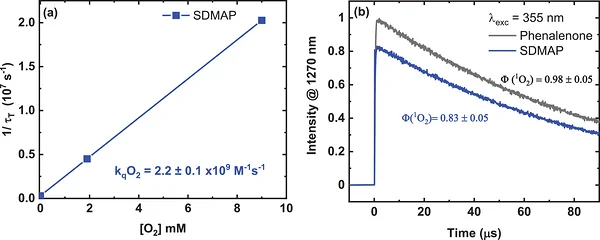
(a) Determination of the bimolecular triplet quenching constants of SDMAP by molecular oxygen. Inverse triplet lifetime versus concentration of molecular oxygen in ACN. (b) Phosphorescence decay traces for singlet oxygen monitored at 1270 nm generated by pulsed photoexcitation at 355 nm of SDMAP and phenalenone in air‐saturated ACN solutions.

Similarly, for SDMAP, following excitation at 600 nm, the positive transient signals with a maximum at 500 nm decay steadily to the baseline within approximately 2 µs, alongside the negative amplitudes at 400 and 562 nm (Figure 7a). In contrast, the broad transient feature observed between 600 and 700 nm remains largely unchanged. As shown in Figure 7a, these dynamics indicate the presence of a long‐lived triplet state in SDMAP, with a lifetime of 0.22 ± 0.01 µs under ambient air conditions. This lifetime decreases notably to 0.049 ± 0.001 µs in oxygen‐saturated environments (Figure S7). To quantify the lifetime of the long‐lived triplet state in the absence of molecular oxygen, we recorded transient data of SDMAP under Ar‐saturated conditions following excitation at 355 nm. As expected, the magnitude of the lifetime increased significantly to 3.40 ± 0.05 µs (see Figure S8 and Table S10). The bimolecular quenching rate constant of the triplet state by oxygen (k_q_O_2_) was calculated as (2.2 ± 0.1) × 10^9^ M^−^^1^s^−^^1^, as shown in Figure 8a. Additionally, nanosecond near‐infrared emission spectroscopy was used to assess singlet oxygen generation. Decay traces of singlet oxygen emission for SDMAP and the phenalenone standard [55] revealed a singlet oxygen quantum yield (Φ_Δ_) of 0.83 ± 0.05 for SDMAP under air‐saturated conditions (see Figure 8b). The kinetic and photophysical properties of DMAP and SDMAP reveal stark contrasts in their electronic relaxation dynamics and singlet oxygen generation efficiencies. DMAP has a relatively short decay lifetime of 5 ns under all conditions of ambient air, O_2_, and N_2_‐saturated, and it does not sensitize singlet oxygen (Φ_Δ_ = 0%) [1]. Conversely, SDMAP exhibits significantly longer triplet lifetimes, reaching up to 3.4 µs under Ar‐saturated conditions, and an impressive singlet oxygen quantum yield of 0.83, indicating efficient energy transfer to oxygen.

### 2.6 Time‐Resolved Fluorescence Spectroscopy

**FIGURE 9:**
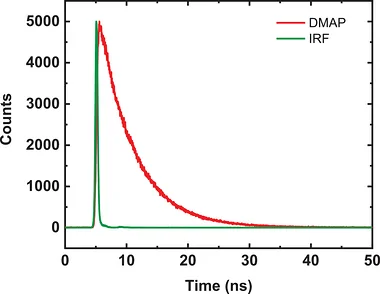
Fluorescence decay profile of DMAP measured at 500 nm in air‐saturated ACN. The IRF curve represents the instrument's response function.

Time‐correlated single‐photon counting (TCSPC) was employed to further characterize DMAP and determine its fluorescence lifetimes in air‐equilibrated ACN. Excitation at 377 nm and monitoring emission at 500 nm revealed a clean monoexponential decay (Figure 9), corresponding to an emitting species with a lifetime of 5.5 ns. This short‐lived emissive component constitutes roughly 17% of the total S_1_‐state population, indicating a minor radiative pathway in DMAP's deactivation to the ground state. Whereas for SDMAP molecule, minimal emission is seen around 730 nm in ACN. The fluorescence decay for SDMAP is negligible as most of the population, rather than decaying back to the ground state through fluorescence, favors population of the triplet state through ISC. This is very well supported by the theoretical calculation, transient absorption spectroscopy. and singlet oxygen quantum yield measurements mentioned earlier for the SDMAP molecule.

### 2.7 Electronic Relaxation Mechanism of DMAP

**SCHEME 2:**
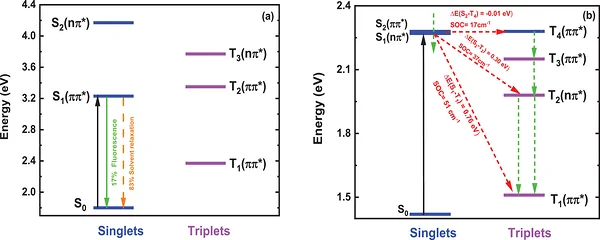
Proposed deactivation mechanisms for (a) DMAP and (b) SDMAP in ACN.

Based on the computational and experimental results outlined in Table 1 and Figure 4 for DMAP, we propose an electronic relaxation mechanism for DMAP in ACN (Scheme 2a). Excitation at 400 nm (3.17 eV) initially populates the S_1_(ππ*) state in the FC region. This is supported by the fact that the second lowest‐energy singlet state (S_2_) primarily exhibits nπ* character with minimal oscillator strength, consistent with its nonfluorescent nature. Following excitation, the femtosecond transient absorption experiments reveal an initial ultrafast decay component (EADS1) with a lifetime of approximately 0.4 ± 0.1 ps, which we attribute to vibrational relaxation within the S_1_(ππ*) PES as it relaxes to its minimum energy structure. This assignment is further supported by the appearance of a transient absorption band at 435 nm, accompanied by a negative amplitude signal at 505 nm (Figure 4a), indicative of excited‐state relaxation. It is also supported by the similar nature of the FC and S_1_ optimized geometries (Figure 3a). Furthermore, the computed ESA profiles for the S_1_(min) state, reported in Figure S9a, exhibit good agreement with the experimental spectra and the EADS1 in Figure 5b.

Given that the T_1_ state lies significantly lower in energy than S_1_ (ΔE(S_1_‐T_1_) = 0.86 eV) and the T_2_ state is nearly isoenergetic with S_1_ (within the ± 0.3 eV error margin of TD‐DFT calculations), ISC could, in principle, compete with radiative decay. However, based on El‐Sayed's rules, ISC between these states is expected to be inefficient due to the lack of a change in orbital character. This is corroborated by the calculated SOC values of 0.01 and 0.04 cm^−^^1^ for the S_1_‐T_1_ and S_1_‐T_2_ transitions, respectively (Table 2), which suggest minimal ISC efficiency. The absence of ISC is further evidenced by the experimentally measured singlet oxygen quantum yield of 0% [1], confirming that population transfer to the triplet manifold is not a significant pathway. A second transient lifetime of 15.0 ± 0.3 ps and the corresponding red EADS2 are attributed to solvent relaxation and solvation dynamics, reflected in the spectral evolution of the transient absorption features.

Finally, nanosecond‐to‐microsecond transient absorption experiments reveal that the excited singlet‐state population of DMAP fully decays back to the ground state with a 5 ± 1 ns lifetime under air, O_2_‐, and N_2_‐saturated conditions in ACN. This rapid decay lifetime aligns with the fluorescence lifetime (5.5 ns) of DMAP (Figure 9). The overall decay of the excited‐state population follows a monoexponential decay behavior governed by the combined radiative and nonradiative process. Given the fluorescence quantum yield of 17%, nonradiative decay dominates the relaxation process. Consistent with the negligible spin‐orbit coupling values (see Tables 2 and S6) and the absence of singlet oxygen generation (Φ_Δ_ = 0%) [1], intersystem crossing to the triplet manifold does not contribute significantly to the excited‐state dynamics.

### 2.8 Electronic Relaxation Mechanism of SDMAP

Based on the quantum chemical calculations and experimental results presented in Figure 4, the ^1^ππ* and ^1^nπ* states of SDMAP are closely coupled and nearly isoenergetic. This near degeneracy could lead to an oscillator strength borrowing if the electronic coupling is strong enough, which could lead to the population of both states. However, according to Table 1, the oscillator strength of the ^1^nπ* state is predicted to be zero. Hence, excitation at 602 nm predominantly populates the ^1^ππ* state due to its higher oscillator strength. A significant portion of the population in the excited S_2_(ππ*) state is anticipated to undergo rapid IC to the S_1_(nπ*) state. Given the energy gap between the relevant singlet and triplet states and substantial SOC values outlined in Table 2, and following El‐Sayed's rules, it is also expected that ISC to the triplet states will proceed through multiple and competing pathways. One pathway involves ISC from the S_2_(ππ*) state directly to the T_2_(nπ*) state, having an energy gap ∆E(S_2_‐T_2_) of 0.30 eV and a SOC value of 37 cm^−1^, followed by IC to the lowest triplet state, T_1_(ππ*). A portion of the S_2_(ππ*) population is also predicted to relax via ultrafast IC to the S_1_(nπ*) state before transitioning to T_1_(ππ*) through ISC. The energy gap ∆E(S_1_‐T_1_) of 0.76 eV and high SOC value of 51 cm^−1^ favor this ISC to occur between S_1_(nπ*) and T_1_(ππ*) states. Finally, another pathway involves ISC from the S_2_(ππ*) state directly to the T_4_(nπ*) state due to the isoenergetic nature of the two states (Table 1), followed by IC from the T_4_(nπ*) to the T_1_(ππ*). Thus, we attribute the initial lifetime of 1.0 ± 0.2 ps, along with the corresponding black EADS1 (see Figure 5d), to a combination of IC from the S_2_(ππ*) state to the S_1_(nπ*) state and ISC from S_1_(nπ*) to T_1_(ππ*) or directly from S_2_(ππ*) to T_2_(nπ*) or T_4_(nπ*). This interpretation is reinforced by the observed transient species, which exhibits a peak absorption at 436 nm, progressively increasing in intensity while becoming more spectrally confined and undergoing a slight red shift. Additionally, this process is accompanied by a transient signal with a negative amplitude near 360 nm, as illustrated in Figure 4b, ii.

The reduction in amplitude of the initial positive band at 436 nm, coupled with a slight blue shift and an increase in intensity of the band at 505 nm, along with the presence of an isosbestic point near 457 nm, is attributed to the vibrationally excited T_1_ state. Therefore, we associate the second lifetime of 93.0 ± 0.3 ps shown as red EADS2 in Figure 5d to the vibrational cooling (VC) and solvent relaxation (SR) within the triplet manifold. The population reaching the triplet state of SDMAP decays within 3.4 µs in N_2_‐saturated conditions. The proposed deactivation mechanisms of DMAP and SDMAP in ACN are presented in Scheme 2.

## 3 Conclusion

Through steady‐state absorption/emission, time‐resolved transient absorption spectroscopy, and nanosecond laser flash photolysis, we investigated the excited‐state dynamics of DMAP and its thionated analog, SDMAP. Theoretical calculations revealed that double thiocarbonyl substitution in SDMAP leads to a substantial bathochromic shift (∼160 nm) of the absorption spectrum into the visible region and increases its SOC values significantly compared to DMAP. The increase in SOC values and the increase in singlet‐to‐triplet relaxation pathways in SDMAP promote efficient ISC to the triplet manifold and explain the remarkable singlet oxygen generation, with near‐unity quantum yield. These properties are crucial for developing efficient photosensitizers with improved tissue penetration in tumor treatment applications. Overall, these findings demonstrate that thionation is an effective strategy for developing low‐cost, HAF‐PSs with enhanced photophysical properties. Thionation of organic carbonyl molecules has significant potential in deep‐tissue PDT, as well as in a wide range of other applications, including bioimaging and photocatalysis.

## Author Contributions

**Chris Acquah**: investigation, formal analysis, writing – original draft, review, and editing. **Sourav K. Seth**: investigation, formal analysis, writing – review and editing. **Eric Lee**: investigation, writing – review and editing. **Steffen Jockusch**: investigation, writing – review and editing. **Reshma Mathew**: investigation, writing – review and editing. **Rubej Khan**: investigation, writing – review and editing. **Shudan Yang**: investigation, writing – review and editing. **Han Xiao**: investigation, writing – review and editing. **Carlos E. Crespo‐Hernández**: conceived the study, funding acquisition, project administration, resources, supervision, visualization, validation, writing – review and editing.

## Conflicts of Interest

The authors declare no conflicts of interest.

## Supporting information




**Supporting File**: chem71137‐sup‐0001‐SuppMat.pdf.
