# Supplementary material for: Double Thionation of 4‐Dimethylaminophthalimide Leads to the Development of a Highly Effective Photosensitizer
Source: Chemistry. 2026 May 18;32(33):e71137. doi: 10.1002/chem.71137 (PMC13532150; doi:10.1002/chem.71137)
Supplement: Supplementary file 1 [file CHEM-32-e71137-s001.pdf]

Double Thionation of 4-Dimethylaminophthalimide Leads to the Development of a Highly Effective Photosensitizer

Chris Acquah,[1] Eric Lee,[1] Sourav Kanti Seth,[1] Reshma Mathew,[1] Rubej Khan,[1] Steffen Jockusch,[2] Shudan Yang,[3] Han Xiao,[3,4,5] & Carlos E. Crespo-Hernández,*[1]

[1] Department of Chemistry, Case Western Reserve University, Cleveland, OH 44106, USA
[2] Center for Photochemical Sciences, Bowling Green State University, Bowling Green, Ohio, 43403, USA
[3] Department of Chemistry, Rice University, 6100 Main Street, Houston, Texas, 77005, USA
[4] Department of Biosciences, Rice University, 6100 Main Street, Houston, Texas, 77005, USA
[5] Department of Bioengineering, Rice University, 6100 Main Street, Houston, Texas, 77005, USA

*Corresponding author; E-mail: carlos.crespo@case.edu, ORCID: 000-0002-3594-0890.

Supporting Information

Experimental and computational methodology

Materials and steady-state measurements

4-dimethylaminophthalimide (DMAP) and thio-4-dimethylaminophthalimide (SDMAP) were obtained and prepared as described by Xiao and co-workers.[1] The synthesis procedure and characterization of the molecules, including ^{1}H NMR, ESI-MS, FT-IR can be found in the cited literature.[1] Both compounds were prepared in HPLC and spectrophotometric grade ACN from Sigma-Aldrich and maintained under dark conditions to prevent degradation. Steady-state absorption and emission spectra were recorded using a Jasko V-130 Spectrophotometer, Cary Eclipse, and Edinburgh Analytical Instruments FLS1000 spectrometers, respectively. Excitation and emission spectra were obtained using a PMT voltage of 600V with 5 nm slit widths and a scan rate of 120 nm/min. Solutions for emission and excitation spectra were prepared with an optical density of ~0.08 at the excitation wavelength of 400 and 602 nm for DMAP and SDMAP, respectively. The fluorescence quantum yield of DMAP was recorded in ACN using Quinine Sulphate (λ_{ex} = 350 nm, Φ_{em} = 0.577) as a reference standard. The standard and corresponding samples were dissolved in 0.1 M H_2SO_4 and ACN, respectively, maintaining an optical density (OD) of 0.05 at the excitation wavelength.

Quantum chemical calculations

The ORCA 5.0.2 software package[2] was used to perform density functional theory (DFT) and time-dependent DFT (TD-DFT) calculations.[3] Ground-state optimizations were performed using the B3LYP/G[4] functional and the def2-TZVP basis set.[5] Bulk implicit solvation effects were modeled using the conductor-like polarizable continuum model (CPCM).[6] VEEs were calculated at the TD-PBE0-D3BJ/CPCM/def2-TZVPD level of theory. The choice of functional for the VEE and SOCs was made based on how well it modeled the HAF-PS under investigation. However, based on our experience with other HAF-PSs and their satisfactory performance, the PBE0[7], CAM-B3LYP[8], and X3LYP[9] were tested first. The frequency calculation was performed using the optimized GS geometry to verify that no imaginary frequencies were observed, ensuring the optimized GS geometry is the local minimum. A split-resolution of identity-j with chain-of-spheres exchange (RIJCOSX)[10] was also applied for all the calculations to reduce the computational cost with negligible cost to accuracy. The def2-TZVP basis set and the CPCM were used in the TD-DFT calculations as well.

Broadband femtosecond transient absorption spectroscopy

The transient absorption setup used has been described in detail in previous publications.[11,12] Briefly, the TAS spectrometer (Helios, Ultrafast Systems) uses a Ti:Sapphire oscillator (Vitesse, Coherent) that seeds a regenerative amplifier (Libra-HE, Coherent), generating pulses of 100 fs (1 kHz and 800 nm). The excitation wavelength was generated by using an optical parametric amplifier (TOPAS, Quantronix/Light Conversion). A translating 2 mm CaF_2 crystal generates the white light continuum, which gives access to a spectral range from 320 to 700 nm to probe the excited state dynamics. During the TAS experiments, the absorbance of the samples at the excitation wavelength was 0.5, and OD matched all runs. The sample was also continually stirred with a Teflon-coated magnetic stir bar to maintain the homogeneity of the solutions. Additionally, samples were frequently refreshed with fresh samples after each scan to minimize contamination of transient signals. A home-made LabView program and Surface Explorer software (Ultrafast Systems) were used for the data collection, while the Glotaran graphical user interface with R-package TIMP software[13] was used for the target data and global analyses. Three sequential kinetic models were used to globally fit the full multidimensional data set for DMAP and SDMAP. Finally, the evolution-associated difference spectra (EADS) were extracted from the global and target analysis.[13]

Nanosecond to microsecond transient absorption spectroscopy

The nanosecond to microsecond transient absorption spectroscopy with picosecond time resolution was conducted using an amplified laser system (Libra-HE, Coherent, Inc., 800 nm, 100 fs, 4.0 W at 1 kHz) to generate the pump pulses. The white light source is described in detail elsewhere.[12] In brief, a photonic

crystal fiber (EOS, Ultrafast Systems, LLC) was employed to generate the broadband white light probe pulses. The electronically triggered white light source delivers an overall time resolution of ca. 400 ps with a repetition rate of 2 kHz and a probe wavelength range of ca. 375 to 780 nm. An excitation wavelength of 400 nm and 600 nm was used for DMAP and SDMAP, respectively. The data were collected with 250 time points over a range of 15 μs. A 2 mm fused silica cuvette was used to acquire data, and the solution was continuously stirred with a Teflon-coated magnetic stirrer. The solution was kept below 4% degradation, as obtained from the absorbance at the excitation wavelength before and after irradiation with the laser for both samples. Measurements were also taken under three atmospheric conditions of ambient air, nitrogen and oxygen, with the latter two being obtained by purging the solution for 15 minutes before data collection.

Singlet quantum yield

The singlet quantum yield of DMAP was already reported elsewhere.[1] Time-resolved luminescence spectroscopy was used to determine the singlet oxygen quantum yields for SDMAP. A Spectra Physics GCR-150-30 Nd:YAG laser (355 nm, 5 ns pulse width) was used as the excitation source for both molecules. Singlet oxygen phosphorescent decay traces were collected at 1270 nm using a modified Fluorolog-3 spectrometer (HORIBA, Jobin Yvon), with a near-IR sensitive photomultiplier tube (H10330A-45, Hamamatsu) as a detector. The decay traces were stored on a digital oscilloscope (TDS 360, Tektronics). The SDMAP solution and the phenalenone standard were prepared in acetonitrile at an optical density of 0.3 at the excitation wavelength in 1 cm path-length quartz cuvettes. The O_2-saturated solutions were bubbled with O_2 gas for at least 20 min before testing. Degradation of the samples was determined to be less than 3% throughout the experiments based on their steady-state absorption spectra. The quantum yields were determined in back-to-back luminescence experiments of SDMAP and Phenalenone solutions under the same conditions, using the reported yield of $^1O_2$ generated by phenalenone as a standard (Φ_Δ = 0.98).[14]

Time-resolved fluorescence measurements

Time-resolved fluorescence measurements of DMAP were performed by time-correlated single photon counting on an Edinburgh Analytical Instruments FLS1000 spectrofluorimeter using a picosecond pulsed-diode laser emitting at 377 nm as excitation source.

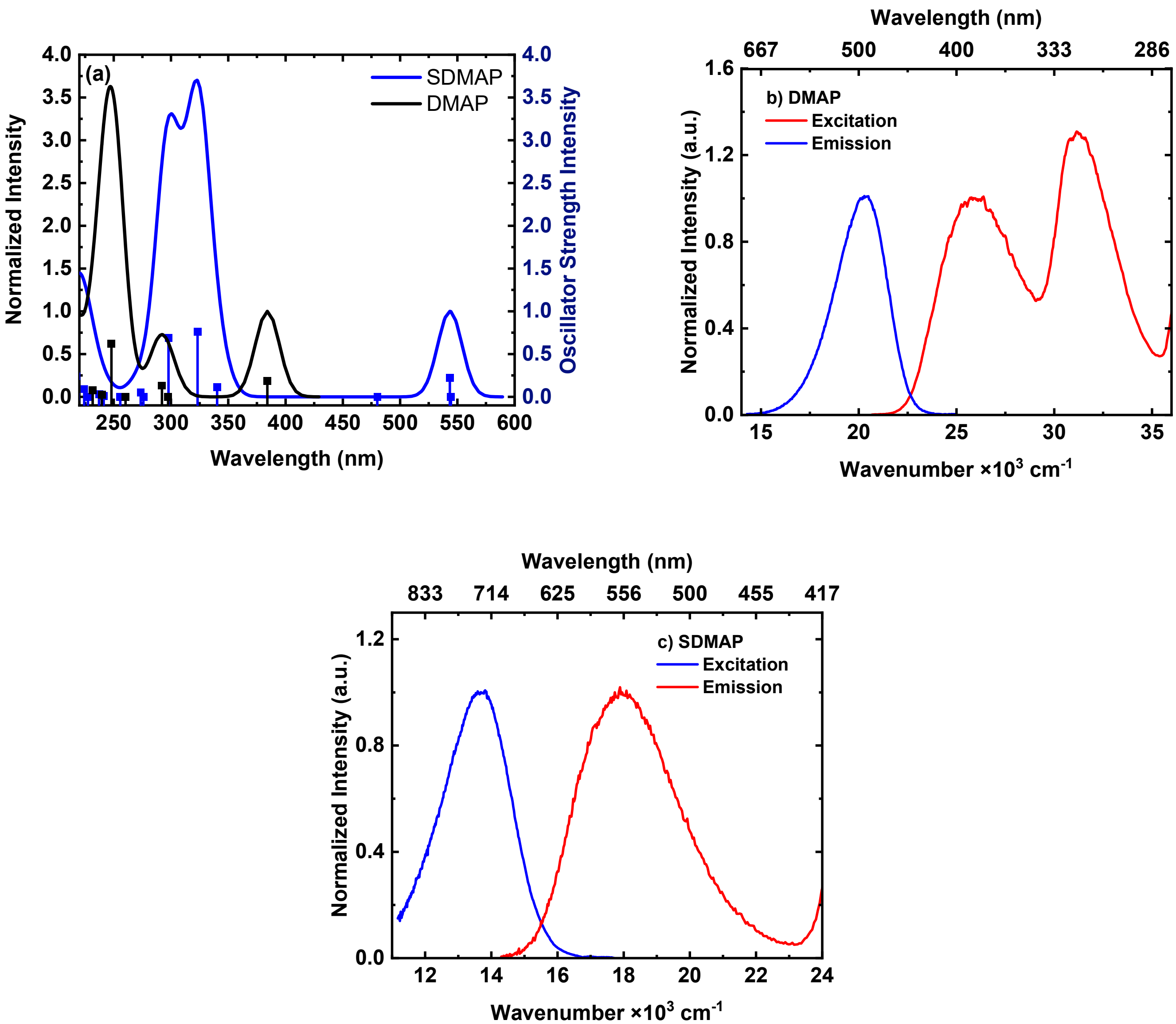

Figure S1. (a) Normalized simulated absorption spectra of DMAP (black) and SDMAP (blue) computed at the TD-PBE0-D3BJ/CPCM/def2-TZVPD//B3LYP$_G$-D3BJ/CPCM/def2-TZVPD level of theory in acetonitrile (ACN). Normalized steady-state excitation and fluorescence emission spectra of (b) DMAP and (c) SDMAP in ACN. Excitation wavelengths of 400 and 550 nm were used for recording the emission spectra of DMAP and SDMAP, respectively.

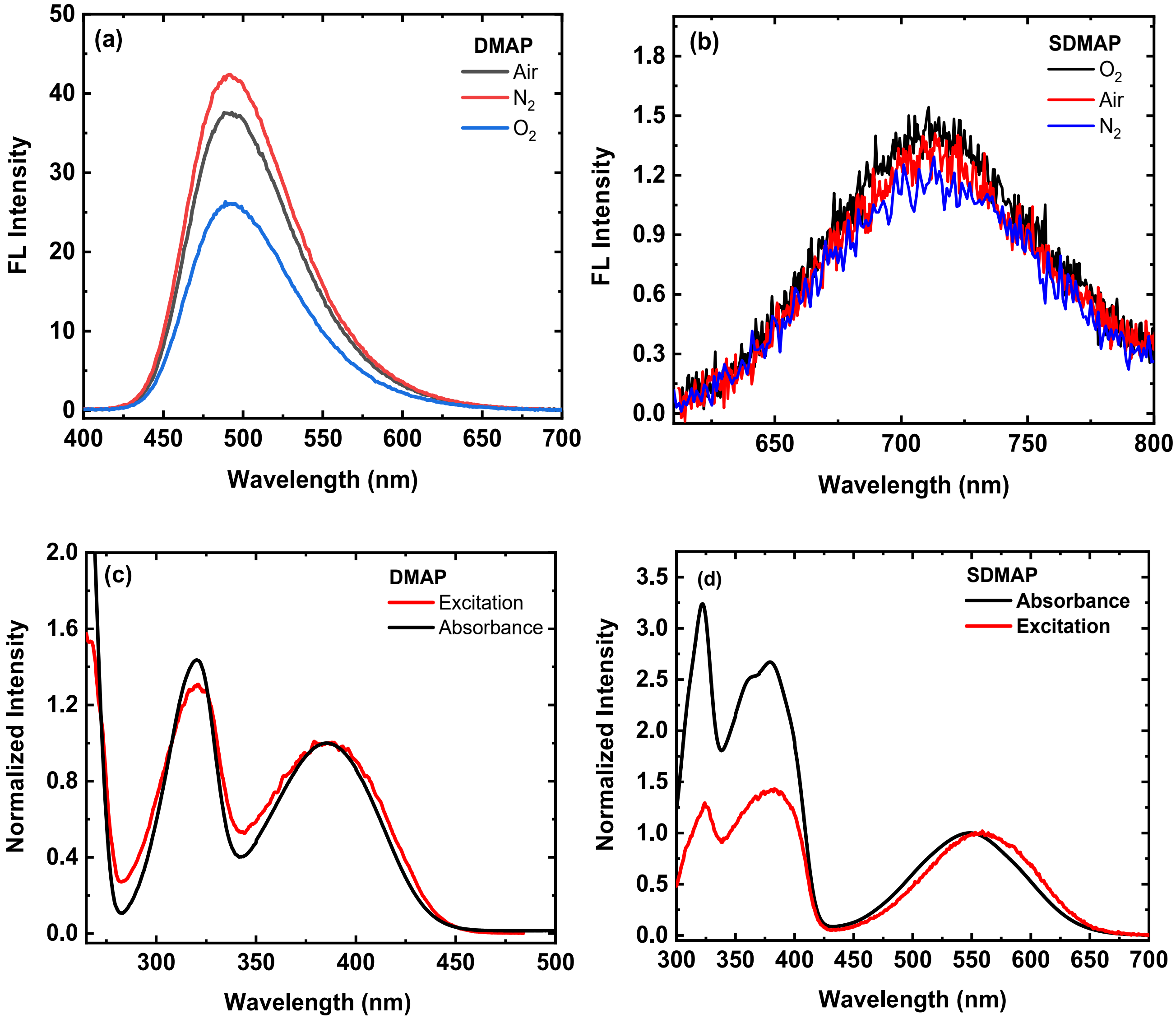

Figure S2. Emission spectra of (a) DMAP and (b) SDMAP in ACN under air-equilibrated, N_2- and O_2-saturated conditions. Absorption and excitation spectra of (c) DMAP and (d) SDMAP in ACN. Excitation spectra with emissions monitored at (c) 494 nm for DMAP and (d) 711 nm for SDMAP.

Table S1. Functional analysis for calculation of vertical excitation energies for the lowest lying singlet and triplet states in the Franck-Condon region for DMAP using the TD-X-D3BJ/CPCM/def2-TZVPD// B3LYP$_G$ -D3BJ/CPCM/def2-TZVPD level of theory (where X = functional) in ACN.

State	CAM-B3LYP	State	X3LYP	State	PBE0
$S_1(\pi\pi^*)$	3.569 (0.207)	$S_1(\pi\pi^*)$	3.147 (0.178)	$S_1(\pi\pi^*)$	3.228 (0.183)
$S_2(\pi\pi^*)$	4.436 (0.135)	$S_2(n\pi^*)$	4.131 (0.000)	$S_2(n\pi^*)$	4.168 (0.000)
$S_3(n\pi^*)$	4.483 (0.000)	$S_3(\pi\pi^*)$	4.174 (0.119)	$S_3(\pi\pi^*)$	4.245 (0.134)
$T_1(\pi\pi^*)$	2.620	$T_1(\pi\pi^*)$	2.331	$T_1(\pi\pi^*)$	2.366
$T_2(\pi\pi^*)$	3.503	$T_2(\pi\pi^*)$	3.340	$T_2(\pi\pi^*)$	3.351
$T_3(\pi\pi^*)$	4.027	$T_3(n\pi^*)$	3.773	$T_3(n\pi^*)$	3.774
$T_4(n\pi^*)$	4.081	$T_4(\pi\pi^*)$	3.831	$T_4(\pi\pi^*)$	3.865

Table S2. Functional analysis for calculation of vertical excitation energies for the lowest lying singlet and triplet states in the Franck-Condon region for SDMAP using the TD-X-D3BJ/CPCM/def2-TZVPD// B3LYP$_G$ -D3BJ/CPCM/def2-TZVPD level of theory (where X = functional) in ACN.

State	CAM-B3LYP	State	X3LYP	State	PBE0
$S_1(n\pi^*)$	2.517 (0.000)	$S_1(n\pi^*)$	2.225 (0.000)	$S_1(n\pi^*)$	2.277 (0.000)
$S_2(\pi\pi^*)$	2.592 (0.263)	$S_2(\pi\pi^*)$	2.232 (0.217)	$S_2(\pi\pi^*)$	2.281 (0.225)
$S_3(n\pi^*)$	2.806 (0.000)	$S_3(n\pi^*)$	2.523 (0.000)	$S_3(n\pi^*)$	2.582 (0.000)
$T_1(\pi\pi^*)$	1.780	$T_1(\pi\pi^*)$	1.496	$T_1(\pi\pi^*)$	1.512
$T_2(n\pi^*)$	2.213	$T_2(n\pi^*)$	1.954	$T_2(n\pi^*)$	1.978
$T_3(\pi\pi^*)$	2.232	$T_3(\pi\pi^*)$	2.164	$T_3(\pi\pi^*)$	2.159
$T_4(n\pi^*)$	2.509	$T_4(n\pi^*)$	2.255	$T_4(n\pi^*)$	2.287

Table S3. Vertical energies and percentage contributions for the relevant singlet and triplet transitions of DMAP calculated at the TD-PBE0-D3BJ/CPCM/def2-TZVPD//B3LYP$_G$-D3BJ/CPCM/def2-TZVPD level of theory in ACN. The primary character of the transition is shown in parentheses.

State	Transition	% Contribution	Energy
**$S_1(\pi\pi^*)$**	H-0 → L+0	93.6	3.23 eV
**$S_2(n\pi^*)$**	H-2 → L+0	96.2	4.17 eV
**$S_3(\pi\pi^*)$**	H-0 → L+1	73.9	4.26 eV
**$T_1(\pi\pi^*)$**	H-0 → L+0	96.6	2.37 eV
**$T_2(\pi\pi^*)$**	H-0→ L+1	94.8	3.35 eV
**$T_3(n\pi^*)$**	H-2 → L+0	93.3	3.77 eV
**$T_4(\pi\pi^*)$**	H-1 → L+0	82.5	3.87 eV

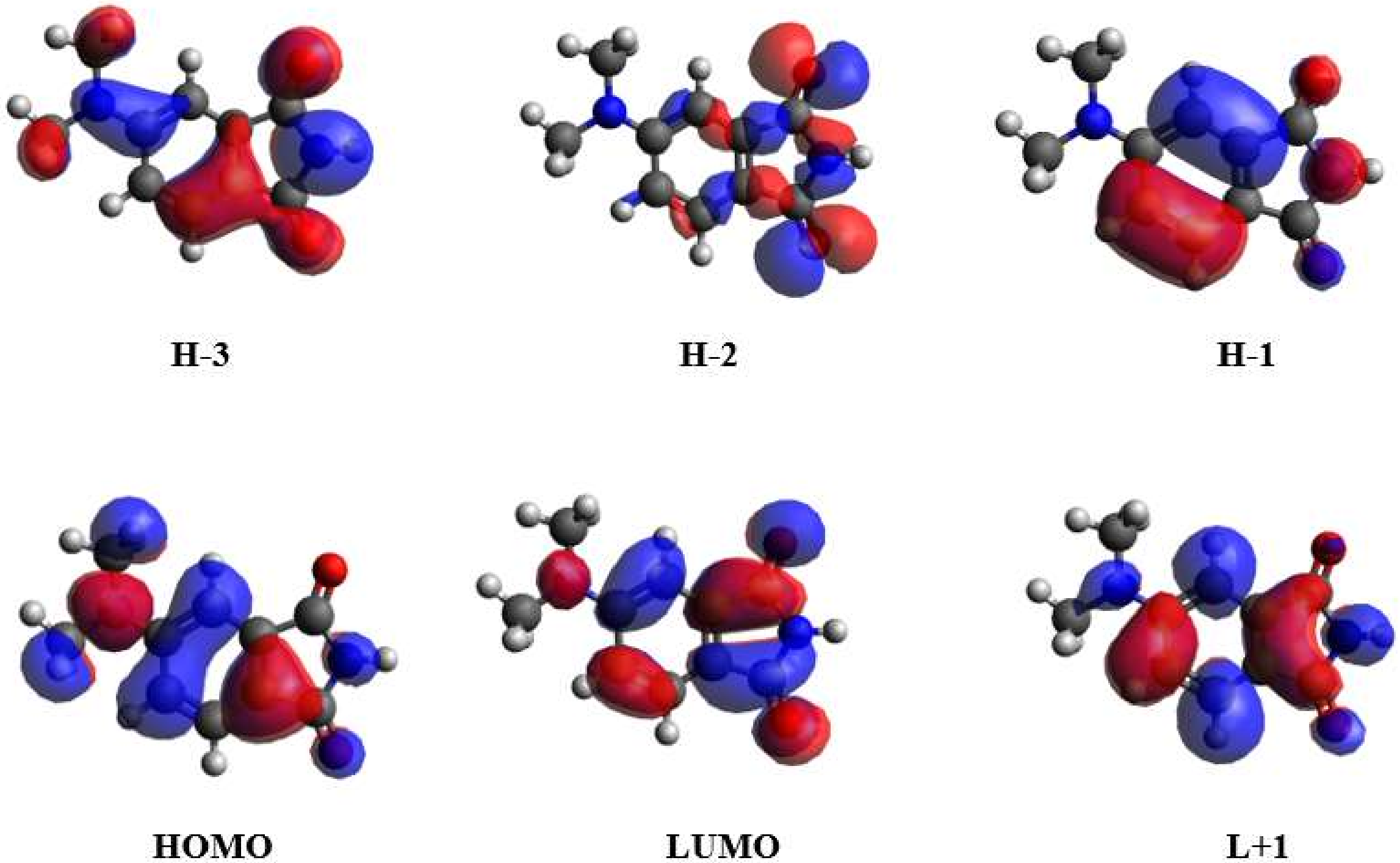

Figure S3. Kohn-Sham orbitals that contribute to the relevant singlet and triplet vertical transitions of DMAP calculated at the TD-CAM-B3LYP-D3BJ/CPCM/def2-TZVPD//B3LYP$_G$-D3BJ/CPCM/def2-TZVPD level of theory in ACN.

Table S4. Vertical energies and percentage contributions for the relevant singlet and triplet transitions of SDMAP calculated at the TD-PBE0-D3BJ/CPCM/def2-TZVPD//B3LYP$_G$-D3BJ/CPCM/def2-TZVPD level of theory in ACN. The primary character of the transition is shown in parentheses.

State	Transition	% Contribution	Energy
**S_1(nπ*)**	H-1 → L+0	96.7	2.27 eV
**S_2(ππ*)**	H-0 → L+0	92.7	2.28 eV
**S_3(nπ*)**	H-2 → L+0	95.9	3.58 eV
**T_1(ππ*)**	H-0 → L+0	97.2	1.51 eV
**T_2(nπ*)**	H-1→ L+0	94.0	1.98 eV
**T_3(ππ*)**	H-3 → L+0	95.7	2.15 eV
**T_4(nπ*)**	H-2 → L+0	92.7	2.29 eV

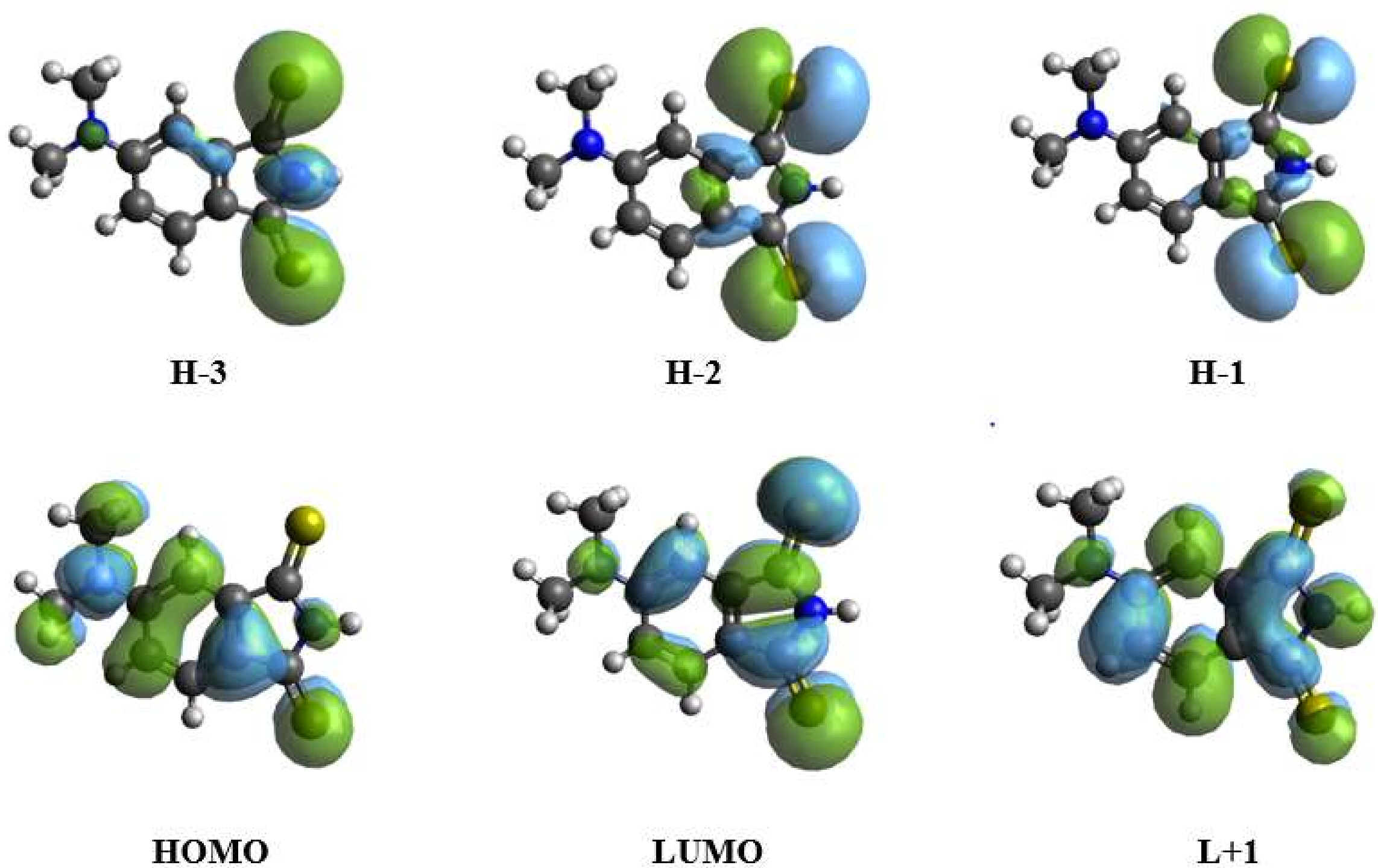

Figure S4. Kohn-Sham orbitals that contribute to the relevant singlet and triplet vertical transitions of SDMAP calculated at the TD-CAM-B3LYP-D3BJ/CPCM/def2-TZVPD//B3LYP$_G$-D3BJ/CPCM/def2-TZVPD level of theory in ACN.

Table S5. Analysis for calculation of vertical excitation energies for the lowest lying singlet and triplet states in the Franck-Condon, S_1, and S_2 regions for DMAP at the TD-PBE0-D3BJ/CPCM/def2-TZVPD// B3LYP$_G$ -D3BJ/CPCM/def2-TZVPD level of theory in ACN.

State	FC	State	S_1 Min	State	S_2 Min
S_1 (ππ*)	3.228 (0.183)	S_1 (ππ*)	2.784 (0.129)	S_1 (ππ*)	2.942 (0.175)
S_2 (nπ*)	4.168 (0.000)	S_2 (nπ*)	3.889 (0.000)	S_2 (nπ*)	3.684 (0.000)
S_3 (ππ*)	4.245 (0.134)	S_3 (ππ*)	4.141 (0.034)	S_3 (ππ*)	4.142 (0.025)
T_1(ππ*)	2.366	T_1(ππ*)	1.960	T_1(ππ*)	2.038
T_2(ππ*)	3.351	T_2(ππ*)	3.327	T_2(nπ*)	3.313
T_3(nπ*)	3.774	T_3(nπ*)	3.506	T_3(ππ*)	3.349
T_4(ππ*)	3.865	T_4(ππ*)	3.524	T_4(ππ*)	3.545
$\Delta E(S_1-T_3)$	-0.546	$\Delta E(S_1-T_3)$	-0.722	$\Delta E(S_1-T_3)$	-0.371
$\Delta E(S_2-T_1)$	1.802	$\Delta E(S_2-T_1)$	1.929	$\Delta E(S_2-T_1)$	1.646
$\Delta E(S_2-T_2)$	0.817	$\Delta E(S_2-T_2)$	0.562	$\Delta E(S_2-T_3)$	0.335
$\Delta E(S_2-T_4)$	0.303	$\Delta E(S_2-T_4)$	0.365	$\Delta E(S_2-T_4)$	0.139
$\Delta E(S_3-T_3)$	0.471	$\Delta E(S_3-T_3)$	0.635	$\Delta E(S_3-T_2)$	0.829

Table S6. Calculated spin-orbit coupling constants for DMAP computed at the TD-PBE0-D3BJ/CPCM/def2-TZVPD//B3LYP$_G$-D3BJ/CPCM/def2-TZVPD level of theory in ACN.

FC Geometry		S_1 Min		S_2 Min	
S_1-T_1 ($^1\pi\pi^*$- $^3\pi\pi^*$)	0.01 cm^{-1}	S_1-T_1 ($^1\pi\pi^*$- $^3\pi\pi^*$)	0.02 cm^{-1}	S_1-T_1 ($^1\pi\pi^*$- $^3\pi\pi^*$)	0.0 cm^{-1}
S_1-T_3 ($^1\pi\pi^*$- $^3n\pi^*$)	7.3 cm^{-1}	S_1-T_3 ($^1\pi\pi^*$- $^3n\pi^*$)	6.1 cm^{-1}	S_1-T_3 ($^1\pi\pi^*$- $^3\pi\pi^*$)	0.04 cm^{-1}
S_2-T_1 ($^1n\pi^*$- $^3\pi\pi^*$)	8.4 cm^{-1}	S_2-T_1 ($^1n\pi^*$- $^3\pi\pi^*$)	7.8 cm^{-1}	S_2-T_1 ($^1n\pi^*$- $^3\pi\pi^*$)	9.5 cm^{-1}
S_2-T_2 ($^1n\pi^*$- $^3\pi\pi^*$)	1.8 cm^{-1}	S_2-T_2 ($^1n\pi^*$- $^3\pi\pi^*$)	4.9 cm^{-1}	S_2-T_2 ($^1n\pi^*$- $^3n\pi^*$)	0.5 cm^{-1}
S_3-T_3 ($^1\pi\pi^*$- $^3n\pi^*$)	2.4 cm^{-1}	S_3-T_3 ($^1\pi\pi^*$- $^3n\pi^*$)	5.0 cm^{-1}	S_3-T_3 ($^1\pi\pi^*$- $^3\pi\pi^*$)	0.02 cm^{-1}

Table S7. Analysis for calculation of vertical excitation energies for the lowest lying singlet and triplet states in the Franck-Condon, S_1, and S_2 regions for for SDMAP at the TD-PBE0-D3BJ/CPCM/def2-TZVPD// B3LYP$_G$ -D3BJ/CPCM/def2-TZVPD level of theory in ACN.

State	FC	State	S1 Min	State	S2 Min
S_1 ($n\pi^*$)	2.277 (0.000)	S_1 ($\pi\pi^*$)	1.996 (0.153)	S_1 ($n\pi^*$)	2.105 (0.000)
S_2 ($\pi\pi^*$)	2.281 (0.225)	S_2 ($n\pi^*$)	2.169 (0.000)	S_2 ($\pi\pi^*$)	2.176 (0.215)
S_3 ($n\pi^*$)	2.582 (0.000)	S_3 ($n\pi^*$)	2.428 (0.000)	S_3 ($n\pi^*$)	2.401 (0.000)
T_1($\pi\pi^*$)	1.512	T_1($\pi\pi^*$)	1.299	T_1($\pi\pi^*$)	1.367
T_2($n\pi^*$)	1.978	T_2($n\pi^*$)	1.869	T_2($n\pi^*$)	1.813
T_3($\pi\pi^*$)	2.159	T_3($\pi\pi^*$)	2.999	T_3($\pi\pi^*$)	1.931
T_4($n\pi^*$)	2.287	T_4($n\pi^*$)	2.135	T_4($n\pi^*$)	2.114
$\Delta E(S_1-T_1)$	0.765	$\Delta E(S_1-T_2)$	0.127	$\Delta E(S_1-T_1)$	0.738
$\Delta E(S_1-T_3)$	0.118	$\Delta E(S_1-T_4)$	-0.139	$\Delta E(S_1-T_3)$	0.174
$\Delta E(S_2-T_2)$	0.303	$\Delta E(S_2-T_1)$	0.870	$\Delta E(S_2-T_2)$	0.363
$\Delta E(S_2-T_4)$	-0.006	$\Delta E(S_2-T_3)$	0.170	$\Delta E(S_2-T_4)$	0.062
$\Delta E(S_3-T_1)$	1.070	$\Delta E(S_3-T_1)$	1.129	$\Delta E(S_3-T_1)$	1.034
$\Delta E(S_3-T_3)$	0.423	$\Delta E(S_3-T_3)$	0.429	$\Delta E(S_3-T_3)$	0.470

Table S8. Calculated spin-orbit coupling constants for SDMAP computed at the TD-PBE0-D3BJ/CPCM/def2-TZVPD//B3LYP$_G$-D3BJ/CPCM/def2-TZVPD level of theory in ACN.

FC Geometry		S_1 Min		S_2 Min	
S_1-T_1 ($^1n\pi^*$- $^3\pi\pi^*$)	51 cm^{-1}	S_1-T_2 ($^1\pi\pi^*$- $^3n\pi^*$)	29 cm^{-1}	S_1-T_1 ($^1n\pi^*$- $^3\pi\pi^*$)	53 cm^{-1}
S_1-T_3 ($^1n\pi^*$- $^3\pi\pi^*$)	63 cm^{-1}	S_1-T_4 ($^1\pi\pi^*$- $^3n\pi^*$)	16 cm^{-1}	S_1-T_3 ($^1n\pi^*$- $^3\pi\pi^*$)	70 cm^{-1}
S_2-T_2 ($^1\pi\pi^*$- $^3n\pi^*$)	37 cm^{-1}	S_2-T_1 ($^1n\pi^*$- $^3\pi\pi^*$)	46 cm^{-1}	S_2-T_2 ($^1\pi\pi^*$- $^3n\pi^*$)	36 cm^{-1}
S_2-T_4 ($^1\pi\pi^*$- $^3n\pi^*$)	17 cm^{-1}	S_2-T_3 ($^1n\pi^*$- $^3\pi\pi^*$)	60 cm^{-1}	S_2-T_4 ($^1\pi\pi^*$- $^3n\pi^*$)	18 cm^{-1}
S_3-T_1 ($^1n\pi^*$- $^3\pi\pi^*$)	42 cm^{-1}	S_3-T_1 ($^1n\pi^*$- $^3\pi\pi^*$)	36 cm^{-1}	S_3-T_1 ($^1n\pi^*$- $^3\pi\pi^*$)	44 cm^{-1}
S_3-T_3 ($^1n\pi^*$-$^3\pi\pi^*$)	148 cm^{-1}	S_3-T_3 ($^1n\pi^*$- $^3\pi\pi^*$)	152 cm^{-1}	S_3-T_3 ($^1n\pi^*$- $^3\pi\pi^*$)	148 cm^{-1}

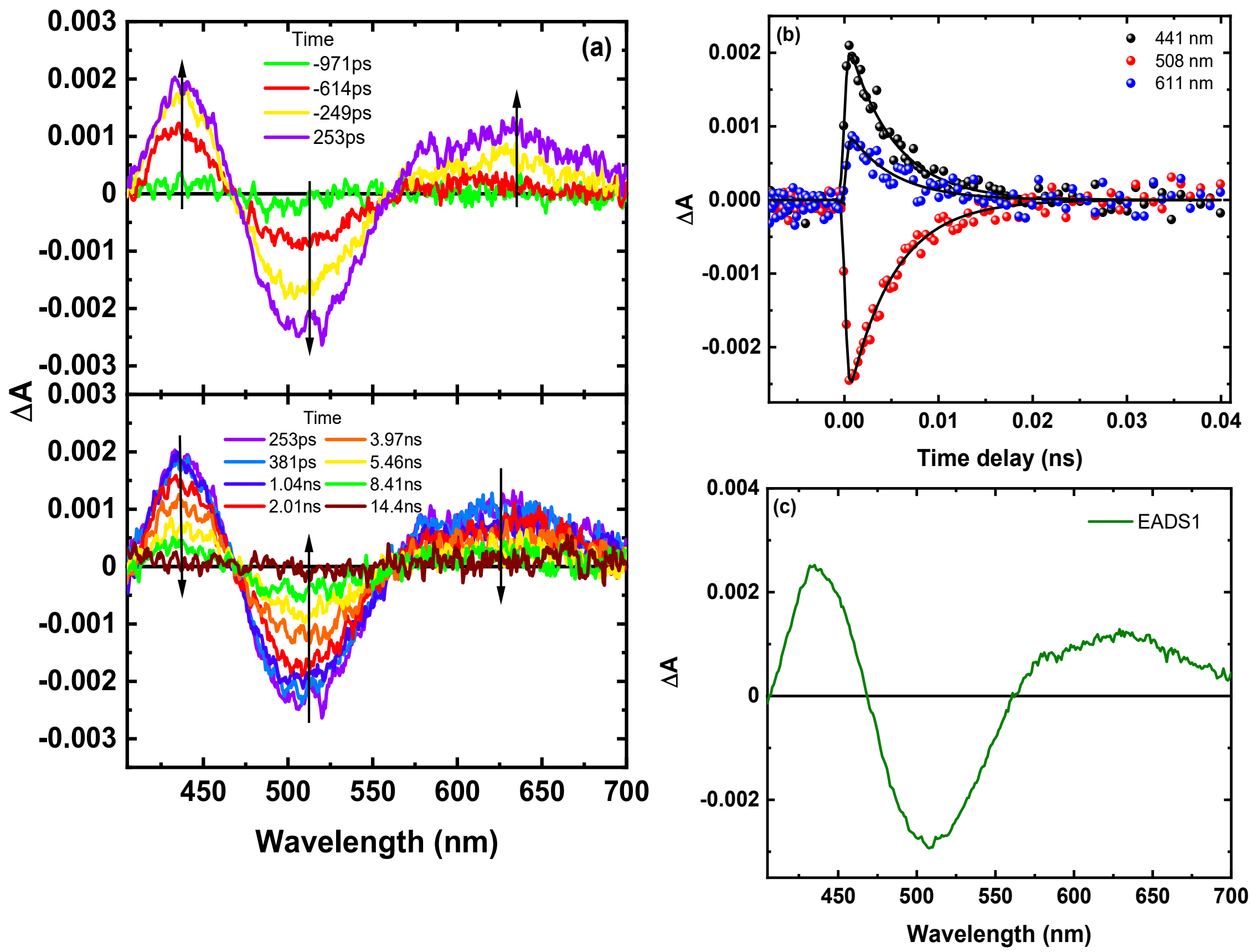

Figure S5. (a) Spectral evolution of the nanosecond transient absorption spectra of DMAP in ACN and following excitation at 400 nm under N_2 saturated conditions. (b) Representative kinetic decay traces and (c) evolution-associated difference spectra obtained from global and target analyses of DMAP in ACN.

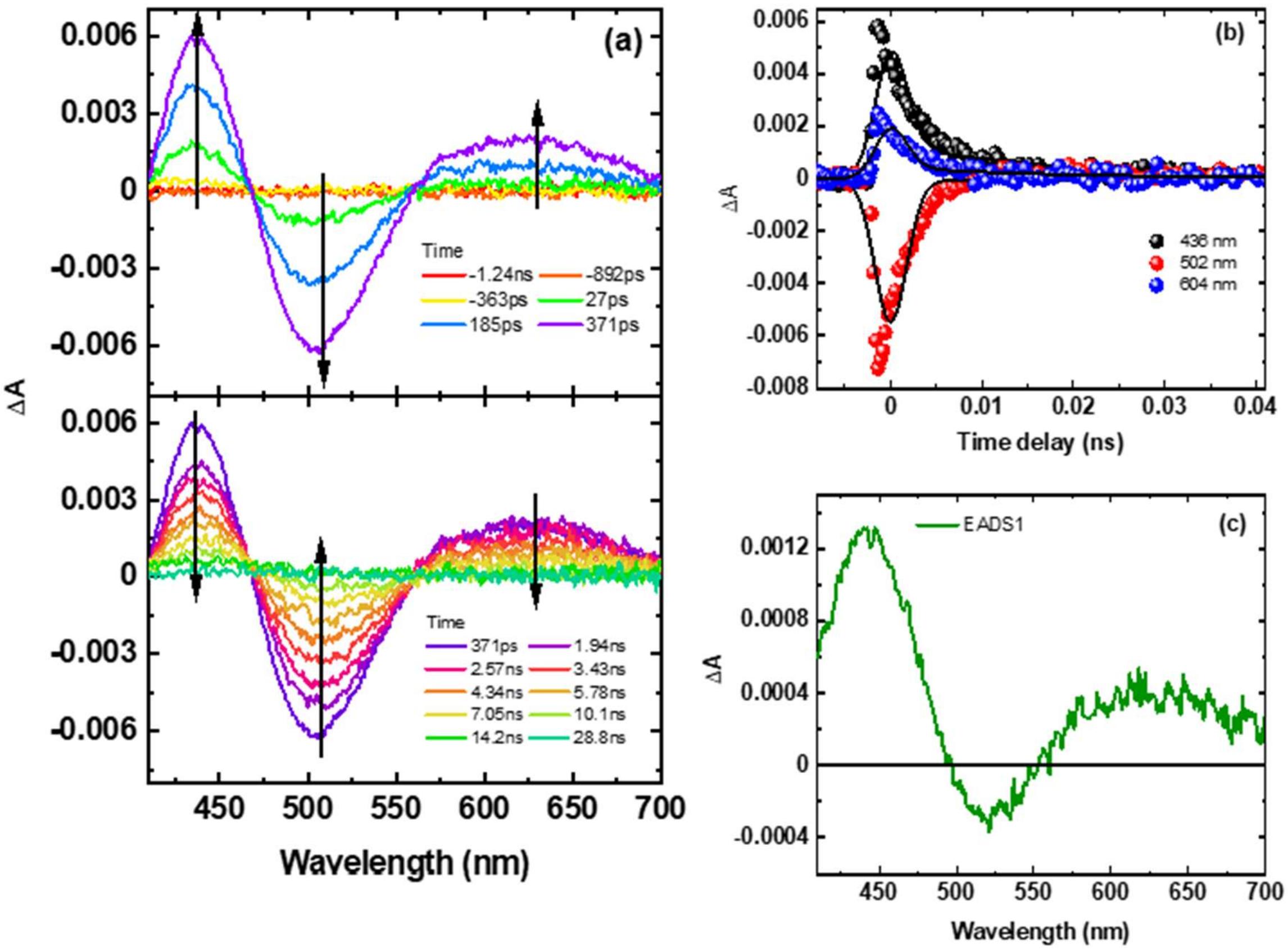

Figure S6. (a) Spectral evolution of the nanosecond transient absorption spectra of DMAP in ACN and following excitation at 400 nm under O_2 saturated conditions. (b) Representative kinetic decay traces and (c) evolution-associated difference spectra obtained from global and target analyses of DMAP in ACN.

Table S9. Global Lifetimes of DMAP following excitation at 400 nm in ACN under Air, O_2 and N_2 saturated conditions. All errors are reported as twice the standard deviation.

	**Lifetime (ns)**
**N_2 saturated**	5 ± 1
**Air Saturated**	5 ± 1
**O_2 saturated**	5 ± 1

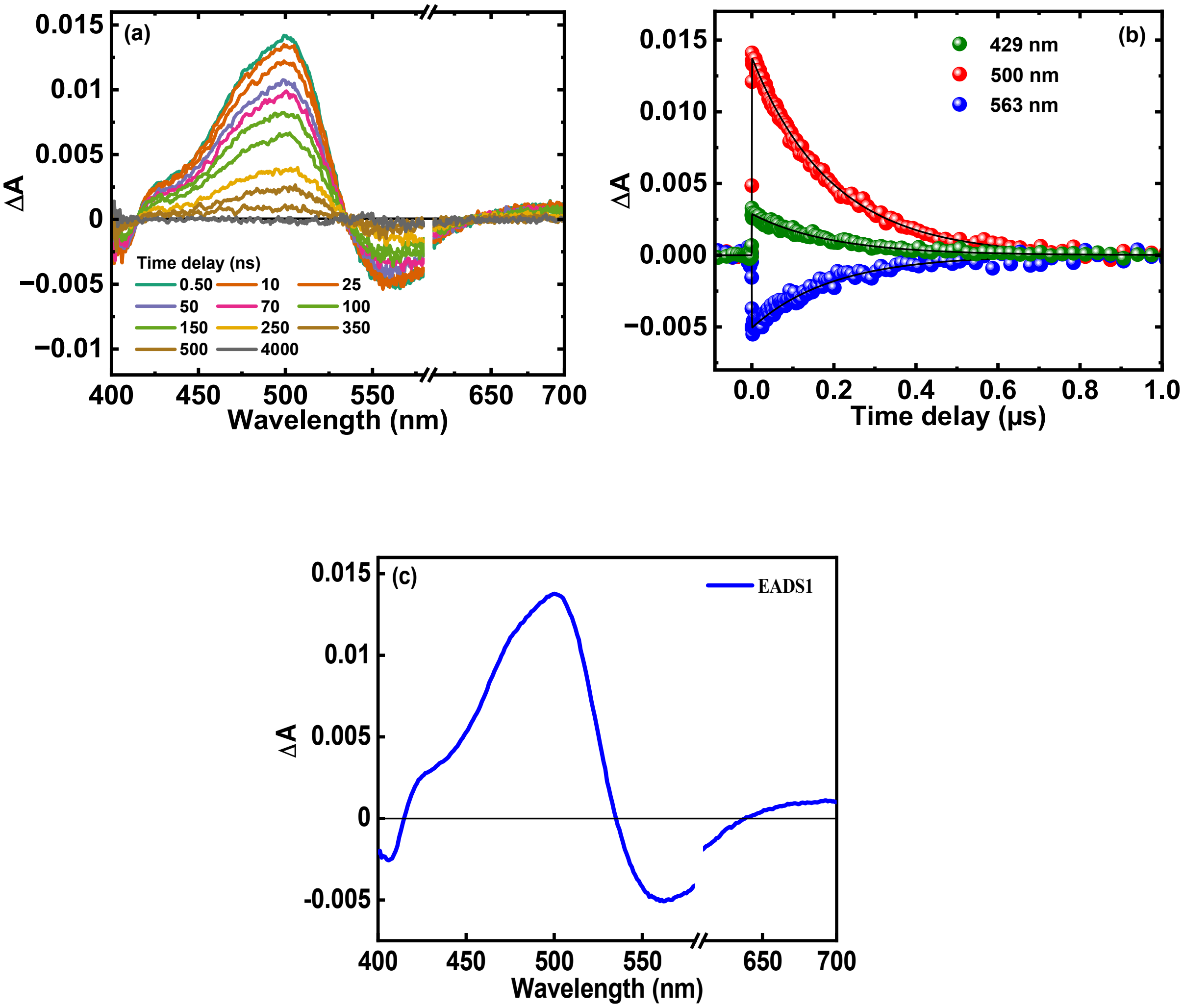

Figure S7. (a) Spectral evolution of the nanosecond transient absorption spectra of SDMAP in ACN and following excitation at 600 nm under N_2 saturated conditions. (b) Representative kinetic decay traces and (c) evolution-associated difference spectra obtained from global and target analyses of SDMAP in ACN.

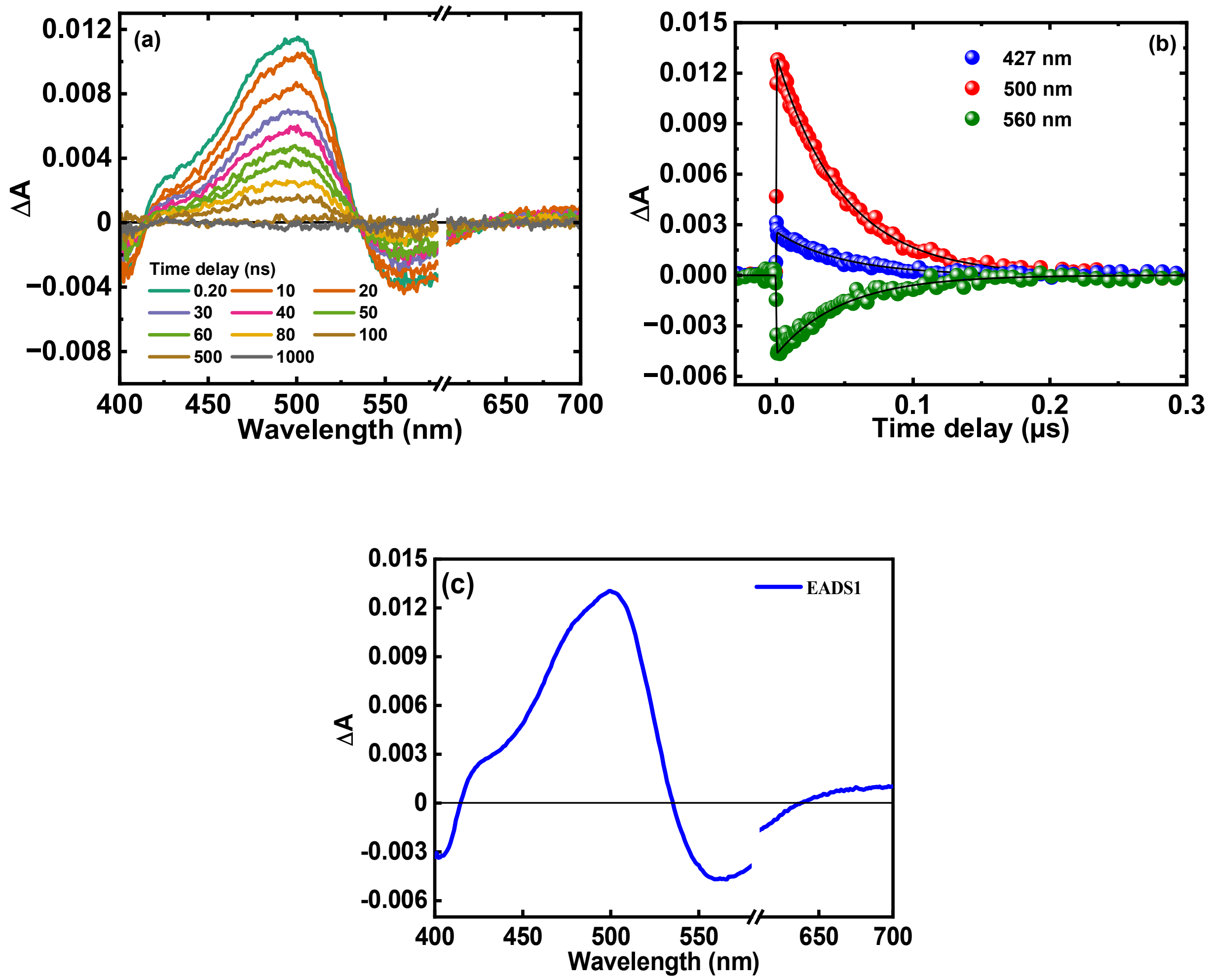

Figure S8. (a) Spectral evolution of the nanosecond transient absorption spectra of SDMAP in ACN and following excitation at 600 nm under O_2 saturated conditions. (b) Representative kinetic decay traces and (c) evolution-associated difference spectra obtained from global and target analyses of SDMAP in ACN.

Table S10. Global Lifetimes of SDMAP following excitation at 600 nm in ACN under Air, O_2 and N_2 saturated conditions. All errors are reported as twice the standard deviation.

	**Lifetime (μs)**
**N_2 saturated**	3.40 ± 0.05
**Air Saturated**	0.22 ± 0.01
**O_2 saturated**	0.049 ± 0.001

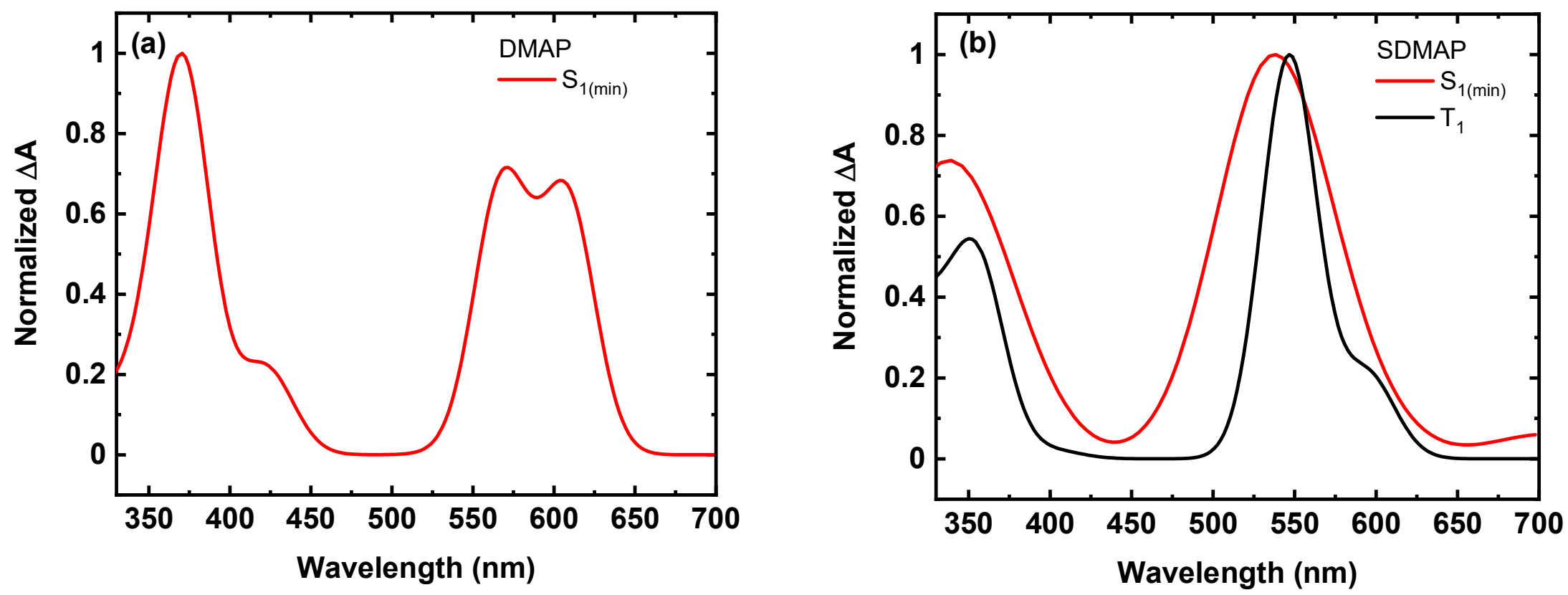

Figure S9. Calculated excited state absorption spectra for the $S_{1(min)}$ and $T_1(\pi\pi^*)$ state of (a) DMAP and (b) SDMAP in ACN.

Cartesian coordinates of relevant states

S_0 DMAP

C	-1.67733605832317	2.43564508226020	0.10677875719289
C	-2.89440496562153	1.69913606208035	0.05503627522631
C	-2.82023053580742	0.27895390331394	0.02308676722774
C	-1.60959686692806	-0.39232126338374	0.03893310616717
C	-0.43525885832274	0.34449789330891	0.08822898665018
C	-0.49634500104463	1.73947920779917	0.12155688450888
N	-4.09197285606546	2.33800189632709	0.03664437562691
C	-4.15176116694425	3.79152335025377	0.06904148694183
H	-3.70089865702555	4.18994832737676	0.98208267472503
H	-5.19180862515972	4.10034978103322	0.03760511620899
H	-3.63567457971729	4.23064755246996	-0.78882064142593
C	-5.33180388622452	1.57893593656397	-0.00801336942600
H	-5.42971806457434	0.92363918644807	0.86176462827386
H	-5.39040178598397	0.96315451116726	-0.90966704723442
H	-6.16743801865316	2.27143291369490	-0.01124797115434
H	-3.72966572611450	-0.29927914053395	-0.01531180482879
H	-1.67338085644094	3.51408769202835	0.13466393299192

H -1.59072308373368 -1.47407306639712 0.01302400019235
C 0.90284336589234 2.25766104451641 0.17524049215060
C 0.97350418157922 -0.06259488560854 0.11677955377315
N 1.70898862604690 1.13194129352981 0.16666381392497
H 2.71780046296231 1.16447436257012 0.19630509989636
O 1.29253197483199 3.40717685837357 0.21778567934625
O 1.47180098137221 -1.17284849919252 0.10230920304406

S_0 SDMAP

C -1.68812636204801 2.43436367043860 0.10621404874313
C -2.90470234630296 1.69736219418471 0.05530278148407
C -2.83215492549733 0.27458275014282 0.02283655445559
C -1.62525232324283 -0.39458682556706 0.03952090754202
C -0.44275251268421 0.33881750071398 0.08921339845578
C -0.50370920249750 1.74375590887773 0.12108810545575
N -4.09824183417785 2.33724683714046 0.03801944494991
C -4.15663123448305 3.79208731880961 0.07463924840591
H -3.70169839933627 4.18468445443766 0.98756289453796
H -5.19634189426542 4.10151214514346 0.04793591364026
H -3.64263077815109 4.23122459066970 -0.78390646642375
C -5.34139459429084 1.58203452357549 -0.01235071159821
H -5.44199431560862 0.92516849523973 0.85538596145608
H -5.39741499144493 0.97011306116336 -0.91630165124082
H -6.17412406181619 2.27764609248884 -0.01577326854358
H -3.74229330609384 -0.30237996277406 -0.01608180295259
H -1.68306931244170 3.51270028221291 0.13319948528595
H -1.60396772077493 -1.47623742265464 0.01392438164867
C 0.88110996491926 2.25012953613631 0.17064129135467
C 0.94203261800568 -0.05664257054381 0.11790733628494
N 1.66664196693189 1.13038743431256 0.16507456877014
H 2.67575239306770 1.15815734931700 0.19307464583365
S 1.42356054427607 3.80205022006815 0.22143438828666
S 1.63645262795699 -1.55461758353355 0.10191854416784

S_1 DMAP

C	-1.68238512976548	2.47714718146283	0.10529880199316
C	-2.86383482883642	1.71781235463517	0.05851275205566
C	-2.81496476123480	0.29155263279340	0.03567971352079
C	-1.60848928023436	-0.37254278999804	0.04952312523608
C	-0.44321079842560	0.37324539680260	0.08872485939155
C	-0.47032483139061	1.79807175749768	0.12402283425897
N	-4.08363284325487	2.32722709537981	0.04018364263875
C	-4.16510897427126	3.76318245175358	0.08283152626343
H	-3.67982150824121	4.14473171339948	0.98739997080836
H	-5.20646534783412	4.06738183997580	0.07518591237416
H	-3.65264075430695	4.20040061789123	-0.78105269310236
C	-5.31518221970469	1.57180359087148	-0.02760560000749
H	-5.40964108779282	0.90471006352611	0.83273705152797
H	-5.35295426218412	0.97183913806684	-0.94039741103469
H	-6.14977598339150	2.26528670881213	-0.02676706353086
H	-3.73201336663367	-0.27873803926930	0.01153971204154
H	-1.70279053997080	3.55730760585541	0.11581133819583
H	-1.57692607891690	-1.45546793914370	0.03115988668581
C	0.88205488885126	2.27066501248724	0.16835846078050
C	0.95866420928098	-0.06850895241193	0.11813480451083
N	1.66927022823923	1.09863613163438	0.16132772474624
H	2.67861282743427	1.12455107026792	0.18887512359054
O	1.35089720502746	3.42403320118874	0.20280432102061
O	1.41971323755706	-1.21475784347891	0.10818120603460

S_1 SDMAP

C	-1.68965923120846	2.47234510285956	0.10978498860083
C	-2.88029991938038	1.71822690544686	0.05985445872466
C	-2.82254656527648	0.28903188636869	0.02777279848483
C	-1.62352675255018	-0.37939860631954	0.04431585198632
C	-0.45562841983638	0.36925538234374	0.09453393280680
C	-0.49117366532298	1.79166059625345	0.13016001292916
N	-4.08838562689082	2.32639416437301	0.04070453661857
C	-4.16744610119065	3.76621545872029	0.07672748829580

H	-3.68742025647894	4.15278653694667	0.98145511359416
H	-5.20933833636283	4.06908161488719	0.06587721637939
H	-3.65661086177534	4.19969286985032	-0.78939598392695
C	-5.32294673325638	1.57125735235887	-0.01887382638238
H	-5.41489001248353	0.90764494045812	0.84445502571178
H	-5.36795352523800	0.97036024188796	-0.93075747366808
H	-6.15585502249971	2.26680746368827	-0.01555922928449
H	-3.73978671076788	-0.28069425193917	-0.00949826416923
H	-1.69914127464443	3.55286051340302	0.12395556530043
H	-1.58912143020303	-1.46160172401685	0.01755770655792
C	0.86275187696734	2.25882727607283	0.17152559393825
C	0.94141479012535	-0.05276265988577	0.11833801641801
N	1.63599434521777	1.10384982752393	0.16176190416378
H	2.64614581580135	1.13280914345918	0.18634470214438
S	1.46724823227016	3.81627728626481	0.21969497235122
S	1.55722538498447	-1.60136732100552	0.09974489242484

S_2 DMAP

C	-1.69151993984078	2.45165504649703	0.10656598657655
C	-2.88404849233167	1.71641841810903	0.05584724658385
C	-2.81376145776419	0.28605721823233	0.02332355962439
C	-1.61914392948598	-0.38535833374828	0.04137325003833
C	-0.42876912161844	0.34632647614491	0.09225949846452
C	-0.48311741729512	1.75806152421502	0.12357778405512
N	-4.09245115282386	2.33908928193614	0.03690071278888
C	-4.15546565034392	3.77930212361222	0.07185829742507
H	-3.69795283287811	4.18103636967841	0.98326518242806
H	-5.19683059825133	4.09095732080729	0.04691155915213
H	-3.64268258696398	4.22592224790582	-0.78762487172844
C	-5.31662191428084	1.57521599343723	-0.01108463536059
H	-5.41928235852588	0.91643514789607	0.85784969577429
H	-5.37662998449328	0.95937867446506	-0.91481475341564
H	-6.15883571584341	2.26291949155087	-0.01465226738995
H	-3.72833765510891	-0.28862896881485	-0.01672385408562

H	-1.69386041976341	3.53219084700795	0.13207724294639
H	-1.60099507379626	-1.46924303864714	0.01574305697907
C	0.86081940205457	2.23992201566460	0.17094516045354
C	0.93588300287795	-0.05609366565253	0.11870595246460
N	1.68539896919466	1.12011369437520	0.16608661415445
H	2.69452345537096	1.15206428875565	0.19342022313936
O	1.36062744900688	3.38732568844088	0.21142995982474
O	1.51210402290442	-1.16149786186894	0.10722939910683

S_2 SDMAP

C	-1.69772541062236	2.44481675253930	0.10872354775123
C	-2.89661633172092	1.71116166212012	0.05845830665884
C	-2.82709170479548	0.28406283455723	0.02506612097059
C	-1.63143162637511	-0.38645358280587	0.03855699526645
C	-0.44002552634046	0.34315350354399	0.08727966282839
C	-0.49495634520706	1.75633501683417	0.12148840977548
N	-4.09869788444048	2.33806552866418	0.04074991748652
C	-4.15941892395717	3.78015184255788	0.07221606364293
H	-3.70202079767119	4.18173353082447	0.98302668097856
H	-5.20053752477609	4.09192912847093	0.04642293308084
H	-3.64643267369057	4.22219516532429	-0.78889830569271
C	-5.32622586404154	1.57829548827089	-0.01155784623038
H	-5.43067074719567	0.91881784715357	0.85626176919781
H	-5.38329095749948	0.96449984399613	-0.91661680392880
H	-6.16596109137314	2.26864428491377	-0.01609545565006
H	-3.74244575780120	-0.28954173216766	-0.01299776283847
H	-1.69797825278401	3.52615363529442	0.13855357572874
H	-1.61358344878944	-1.47083926579872	0.01287112669602
C	0.85323065353604	2.23623236098349	0.17002880485947
C	0.92048547748935	-0.04614207739781	0.11462098565325
N	1.65196664261517	1.11985807155513	0.16396946506327
H	2.66031180770348	1.15043526382112	0.19206854360352
S	1.46655298110445	3.79367647964614	0.22628638238041
S	1.65161330663295	-1.55768158290125	0.09999688271816

T_1 DMAP

C	-1.68057024797834	2.46932711981409	0.10727571577705
C	-2.86683614229244	1.72116031574920	0.05187575023924
C	-2.81434574591156	0.26774131405138	0.01501707437256
C	-1.61901303799633	-0.38840776621485	0.03005966529454
C	-0.43751760507791	0.35808181332718	0.07870659094055
C	-0.47008660969201	1.79602264862488	0.12034627974719
N	-4.07121937132621	2.33106553067867	0.03755832578674
C	-4.15894446866601	3.77657162118001	0.06496296881185
H	-3.68327632908297	4.16901053485335	0.96739014083151
H	-5.20384017590874	4.06984096714388	0.05566650089043
H	-3.65805353597227	4.20377991814730	-0.80778201273151
C	-5.30900275467732	1.57373853560828	-0.00268037517574
H	-5.38654628859266	0.91965817830191	0.86815963125578
H	-5.35984465313731	0.96774404309403	-0.90951742896795
H	-6.14247340296797	2.26884822064177	0.00444302524246
H	-3.73344107818731	-0.29819293257984	-0.02024016912643
H	-1.70197217517047	3.55019909842947	0.13452501491521
H	-1.58659988274179	-1.47159142302806	0.00590943758946
C	0.87581221175440	2.27204774736418	0.17706531133738
C	0.94879268282639	-0.06555787101767	0.11861812300228
N	1.66485744593180	1.11165156192178	0.17307088060555
H	2.67300054476052	1.13983253025443	0.20853235582521
O	1.34023161317865	3.41989238472837	0.22211403486496
O	1.42993900692794	-1.20289409107382	0.10939315867166

T_1 SDMAP

C	-1.68973718768102	2.45882567439887	0.10953132918146
C	-2.88258057155185	1.70894476020082	0.05698551602348
C	-2.82700223143842	0.26669964884797	0.02021740296873
C	-1.63760664453529	-0.39205611587336	0.03581195531043
C	-0.44748426866994	0.35585069735108	0.09038073614249
C	-0.48705470083006	1.78179283419219	0.12882568838837

N	-4.07681073335517	2.32962462059865	0.03985866722333
C	-4.15776224929166	3.77733076066686	0.06789949585201
H	-3.68536542070116	4.16611887667700	0.97314835957747
H	-5.20180175163627	4.07299127302562	0.05451138886025
H	-3.65293034522875	4.20210629214987	-0.80327817271910
C	-5.31698676317958	1.57725737875958	-0.00718330879500
H	-5.39603429268446	0.91996319266494	0.86125264649169
H	-5.36211391406082	0.97362353759468	-0.91620291194861
H	-6.14879064998736	2.27397733271757	-0.00151517450426
H	-3.74556084310109	-0.30047605087106	-0.02057742059730
H	-1.70046024521041	3.53999557277967	0.13086012720817
H	-1.60179043462305	-1.47459888010385	0.00668733046031
C	0.85791315143533	2.25850122946501	0.17672850867946
C	0.92018729663974	-0.04637725452823	0.11713840776817
N	1.63097192873426	1.12379659656163	0.16803222451179
H	2.63966645653989	1.15157661807668	0.19529672378402
S	1.44890949963324	3.82439425828128	0.23145540742234
S	1.56927491478394	-1.59030285363351	0.09461507271037

References

[1] J. Tang, L. Wang, A. Loredo, C. Cole, H. Xiao, "Single-Atom Replacement as a General Approach Towards Visible-Light/Near-Infrared Heavy-atom-free photosensitizers for photodynamic therapy" *Chem. Sci.* **2020**, *11*, 6701–6708.

[2] F. Neese, "The ORCA program system" *Wiley Interdiscip. Rev. Comput. Mol. Sci.* **2012**, *2*, 73–78.

[3] A. D. Becke, "A New Mixing of Hartree–Fock and Local Density-Functional Theories" *J. Chem. Phys.* **1993**, *98*, 1372–1377.

[4] S. Tsuzuki, T. Uchimaru, "Accuracy of Intermolecular Interaction Energies, Particularly those of Hetero-Atom Containing Molecules Obtained by DFT Calculations with Grimme's D2, D3 and D3BJ Dispersion Corrections" *Phys. Chem. Chem. Phys.* **2020**, *22*, 22508–22519.

[5] F. Weigend, R. Ahlrichs, "Balanced Basis Sets of Split Valence, Triple Zeta Valence and Quadruple Zeta Valence Quality for H to Rn: Design and Assessment of Accuracy" *Phys. Chem. Chem. Phys.* **2005**, *7*, 3297–3305.

[6] V. Barone, M. Cossi, "Quantum Calculation of Molecular Energies and Energy Gradients in Solution by a Conductor Solvent Model" *J. Phys. Chem. A.* **1998**, *102*, 1995–2001.

[7] C. Adamo, V. Barone, "Toward Reliable Density Functional Methods Without Adjustable Parameters: The PBE0 Model" *J. Chem. Phys.* **1999**, *110*, 6158–6170.

[8] T. Yanai, D. P. Tew, N. C. Handy, "A New Hybrid Exchange–Correlation Functional Using the Coulomb-Attenuating Method (CAM-B3LYP)" *Chem. Phys. Lett.* **2004**, *393*, 51–57.

[9] X. Xu, W. A. Goddard, "The X3LYP Extended Density Functional for Accurate Descriptions of Nonbond Interactions, Spin States, and Thermochemical Properties" *Proc. Natl. Acad. Sci.* **2004**, *101*, 2673–2677.

[10] F. Neese, F. Wennmohs, A. Hansen, U. Becker, "Efficient, Approximate and Parallel Hartree–Fock and Hybrid DFT Calculations. A 'Chain-of-Spheres' Algorithm for the Hartree–Fock Exchange, *Chem. Phys.* **2009**, *356*, 98–109.

[11] C. Reichardt, C. E. Crespo-Hernaíndez, "Room-Temperature Phosphorescence of the DNA Monomer Analogue 4-thiothymidine in Aqueous Solutions after UVA Excitation" *J. Phys. Chem. Lett.* **2010**, *1*, 2239–2243.

[12] C. Reichardt, R. A. Vogt, C. E. Crespo-Hernández, "On the Origin of Ultrafast Nonradiative Transitions in Nitro-polycyclic Aromatic Hydrocarbons: Excited-state Dynamics in 1-Nitronaphthalene" *J. Chem. Phys.* **2009**, *131*, 224518

[13] J. J. Snellenburg, S. Laptenok, R. Seger, K. M. Mullen, I. H. M. van Stokkum, ""Glotaran: A Java-Based Graphical User Interface for the R Package TIMP,"" *J Stat Softw* **2012**, *49*, 1–22.

[14] A. M. Brouwer, "Standards for Photoluminescence Quantum Yield Measurements in Solution (IUPAC Technical Report)" *Pure Appl. Chem.* **2011**, *83*, 2213–2228.
